# *i*PASTIC: An online toolkit to estimate plant abiotic stress indices

**DOI:** 10.1002/aps3.11278

**Published:** 2019-07-17

**Authors:** Alireza Pour‐Aboughadareh, Mohsen Yousefian, Hoda Moradkhani, Mohammad Moghaddam Vahed, Peter Poczai, Kadambot H. M. Siddique

**Affiliations:** ^1^ Seed and Plant Improvement Institute Agricultural Research, Education and Extension Organization (AREEO) Karaj Iran; ^2^ Department of Computer Science University of Manitoba Winnipeg Manitoba Canada; ^3^ Department of Plant Breeding Kermanshah Branch Islamic Azad University Kermanshah Iran; ^4^ Department of Plant Breeding and Biotechnology University of Tabriz Tabriz Iran; ^5^ Botany Unit Finnish Museum of Natural History University of Helsinki P.O. Box 7 Helsinki FI‐00014 Finland; ^6^ Department of Molecular Plant Physiology Institute for Water and Wetland Research Radboud University 6500 GL Nijmegen The Netherlands; ^7^ The UWA Institute of Agriculture The University of Western Australia LB 5005 Perth Western Australia 6001 Australia

**Keywords:** abiotic stresses, online software, principal component analysis, selection index, three‐dimensional plot, tolerance and susceptibility indices

## Abstract

**Premise:**

In crop breeding programs, breeders use yield performance in both optimal and stressful environments as a key indicator for screening the most tolerant genotypes. During the past four decades, several yield‐based indices have been suggested for evaluating stress tolerance in crops. Despite the well‐established use of these indices in agronomy and plant breeding, a user‐friendly software that would provide access to these methods is still lacking.

**Methods and Results:**

The Plant Abiotic Stress Index Calculator (*i*PASTIC) is an online program based on JavaScript and R that calculates common stress tolerance and susceptibility indices for various crop traits including the tolerance index (TOL), relative stress index (RSI), mean productivity (MP), harmonic mean (HM), yield stability index (YSI), geometric mean productivity (GMP), stress susceptibility index (SSI), stress tolerance index (STI), and yield index (YI). Along with these indices, this easily accessible tool can also calculate their ranking patterns, estimate the relative frequency for each index, and create heat maps based on Pearsonʼs and Spearmanʼs rank‐order correlation analyses. In addition, it can also render three‐dimensional plots based on both yield performances and each index to separate entry genotypes into Fernandezʼs groups (A, B, C, and D), and perform principal component analysis. The accuracy of the results calculated from our software was tested using two different data sets obtained from previous experiments testing the salinity and drought stress in wheat genotypes, respectively.

**Conclusions:**

*i*PASTIC can be widely used in agronomy and plant breeding programs as a user‐friendly interface for agronomists and breeders dealing with large volumes of data. The software is available at https://mohsenyousefian.com/ipastic/.

The climate crisis has increased the frequency and intensity of both abiotic and biotic stressors. In recent decades, the effects of these stressors on crop production have become increasingly important (Vaughan et al., 2018). The efficiency of breeding programs in diverse environments can be significantly improved by gaining an understanding of the associations between yield performance and different selection criteria, as well as by accurately estimating stress tolerance in genetic materials (Collard and Mackill, 2008; Xu, 2016). In most crops, yield performance is the main criterion for evaluating tolerance to different environmental stressors. For example, in crop improvement programs, breeders use yield performance and its stability under different growth conditions (e.g., drought, salinity, temperature extremes, and biotic stressors) as a major indicator of stress tolerance. Therefore, screening for tolerance to a specific stress is based on high performance in non‐stressed and stressed environments (Clarke et al., 1992), such that genotypes with high yields in both environments are considered tolerant.

Based on Fernandezʼs theory (Fernandez, 1992), genotypes can be categorized into four groups, based on their yield response to stressful conditions: (1) relatively uniform performance in both non‐stressed and stressed environments (Group A), (2) high performance in non‐stressed environments (Group B), (3) high performance in stressed environments (Group C), and (4) low performance in both non‐stressed and stressed environments (Group D). In relation to these classifications, several yield‐based stress tolerance and susceptibility indices have been formulated to characterize the response of genotypes in different environments, and to select for tolerant genotypes. These are: tolerance index (TOL; Rosielle and Hamblin, 1981), relative drought index (RDI; Fischer and Wood, 1979; herein referred to as relative stress index [RSI]), mean productivity (MP; Rosielle and Hamblin, 1981), harmonic mean (HM; Bidinger et al., 1987), yield stability index (YSI; Bouslama and Schapaugh, 1984), geometric mean productivity (GMP; Fernandez, 1992), stress susceptibility index (SSI; Fischer and Maurer, 1978), stress tolerance index (STI; Fernandez, 1992), and yield index (YI; Gavuzzi et al., 1997).

The nine proposed indices were first used to screen for drought‐tolerant genotypes and are more commonly known as drought‐stress indices. Nonetheless, these indices can be used in other studies—including those of abiotic and biotic stressors—for screening tolerant and susceptible genotypes. During the past four decades, these indices have been developed and used independently in numerous breeding programs. However, a software package that amalgamates all of these indices into a single source has thus far not been developed. Therefore, we offer the first user‐friendly online software that meets this need, the Plant Abiotic Stress Index Calculator (*i*PASTIC).

## METHODS AND RESULTS

### Description of *i*PASTIC software and its functionalities

**Figure 1:**
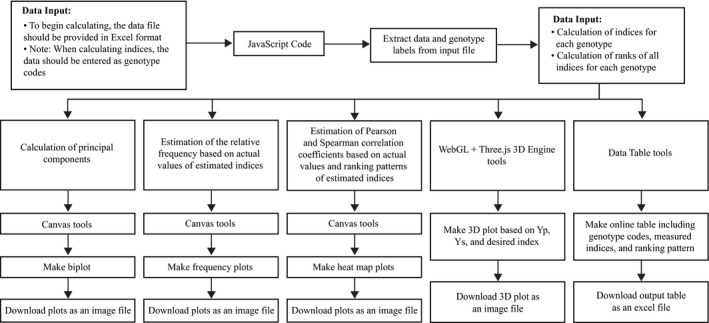
Information flow diagram for *i*PASTIC software.

Table 1 shows the mathematical formulas and selection pattern for each index. *i*PASTIC is written in the JavaScript programming language on the browser‐side and PHP on the server‐side, and is available as a web application (https://mohsenyousefian.com/ipastic/). Alternatively, users can access the source codes in R language (R Development Core Team, 2014) and supporting data sets on GitHub (https://github.com/pour-aboughadareh/iPASTIC/). In addition to the web application, *i*PASTIC is available in R language for more advanced users. Figure 1 shows the information flow of this software. The software reads standard Microsoft Excel formats, hence it is easy and approachable even for users with limited knowledge of computer programming languages. As its core functionality, *i*PASTIC calculates the nine indices and the percentage of relative change due to stress relative to the non‐stress environment for a set of genotypes. It also calculates the ranking patterns of the genotypes, based on each index. Using the WebGL and Three.js frameworks (Cabello, 2014), this software renders an interactive three‐dimensional (3D) plot based on yield (Yp: yield performance under non‐stressed conditions, and Ys: yield performance under stressed conditions) for each index. As a result, users can assign the genotypes to groups A, B, C, and D, as described by Fernandez (1992). Based on Pearsonʼs and Spearmanʼs rank‐order correlation coefficients (Pearson, 1895; Spearman, 1904), *i*PASTIC can identify interrelationships among the indices and their ranks using heat map(s), which are displayed with the Canvas tool. The relative frequency of each index can also be estimated. Principal component analysis (PCA) is another tool available in this software, which enables users to visualize the associations between the tested genotypes and index vectors in a PCA‐based biplot.

**Table 1:** Mathematical formulas of tolerance and susceptibility indices calculated by *i*PASTIC software

Index	Formula	Pattern of selection	Reference
Tolerance	TOL = Y_P_ − Y_S_	Minimum value	Rosielle and Hamblin (1981)
Mean productivity	$\text{MP}=\frac{{\text{Y}}_{P}+{\text{Y}}_{S}}{2}$	Maximum value	Rosielle and Hamblin (1981)
Geometric mean productivity	$\text{GMP}=\sqrt{{\text{Y}}_{S}\times {\text{Y}}_{P}}$	Maximum value	Fernandez (1992)
Harmonic mean	$\text{HM}=\frac{2({\text{Y}}_{S}\times {\text{Y}}_{P})}{\left({\text{Y}}_{S}+{\text{Y}}_{P}\right)}$	Maximum value	Bidinger et al. (1987)
Stress susceptibility index	$\text{SSI}=\frac{1-({\text{Y}}_{S}/{\text{Y}}_{P})}{1-({\overset{¯}{\text{Y}}}_{S}/{\overset{¯}{\text{Y}}}_{P})}$	Minimum value	Fischer and Maurer (1978)
Stress tolerance index	$\text{STI}=\frac{{\text{Y}}_{S}\times {\text{Y}}_{P}}{{\left({\overset{¯}{\text{Y}}}_{P}\right)}^{2}}$	Maximum value	Fernandez (1992)
Yield index	$\text{YI}=\frac{{\text{Y}}_{S}}{{\overset{¯}{\text{Y}}}_{s}}$	Maximum value	Gavuzzi et al. (1997)
Yield stability index	$\text{YSI}=\frac{{\text{Y}}_{S}}{{\text{Y}}_{P}}$	Maximum value	Bouslama and Schapaugh (1984)
Relative stress index	$\text{RSI}=\frac{\left({\text{Y}}_{S}/{\text{Y}}_{P}\right)}{\left({\overset{¯}{\text{Y}}}_{S}/{\overset{¯}{\text{Y}}}_{P}\right)}$	Maximum value	Fischer and Wood (1979)

After following the instructions on the website, the results are displayed in five separate tabs. The first tab, *Indices*, includes two separate sheets. The first sheet displays average yield (for each trait) under non‐stressful and stressful conditions, relative change due to stress, and actual values of the nine measured indices. The second sheet displays genotype rankings for each index, along with sum ranks, average sum of ranks (ASR), and standard deviation (SD), all of which are downloadable in Excel format.

The second tab, *Frequencies*, provides the relative frequency of genotypes based on yield and each index. This tab enables users to obtain more information regarding the distribution of the genotypes into different classes. When one index is selected, the genotypes belonging to each class are displayed at the bottom of the frequency plot.

In the third tab, *Correlation Plots*, associations among different indices and yield are shown in two distinct heat maps. Pearsonʼs correlation analysis estimates the correlation coefficients of the correlation among the actual values of the indices; Spearmanʼs rank‐order correlation analysis shows the relationships among the ranks of the indices. The user has the option of displaying the results as one of three different heat maps (i.e., square, circle, or mixed values and circle).

The fourth tab, *Three‐dimensional*, renders a 3D plot for each index along with yield. The third dimension is adjustable, and users can select any of the indices from the menu bar at the bottom of the page to create a 3D plot. *i*PASTIC also has a tool to check the position of each genotype individually. Selecting one or more genotypes in the “Genotypes control panel” on the right side of the indexʼs menu bar will display the position of the selected genotypes. Clicking on the bars in the 3D plot will display the label for each genotype. The viewing angle of the plot can be changed by dragging the 3D plot. After selecting the best position and viewing angle, the final graph can be downloaded as an image file.

The fifth tab, *PCA*, shows the results of the PCA analysis, which is mostly used as a multivariate approach in investigative data analysis and for predictive models. It can also be used to visualize distance and relatedness between entries. PCA can be done by eigenvalue decomposition of a data correlation (or covariance) matrix, or singular value decomposition of a data matrix, usually after a normalization step of the initial data. The results of PCA are downloaded in an Excel file. In the output file, the summary of descriptive statistics (including minimum, maximum, mean, and SD), correlation (or covariance) matrix, eigenvalues, eigenvectors, factor loading, contribution of variables in each component, and factor scores for each genotype are displayed in separate sheets. In this section, the biplot is initially rendered on the first two principal components, but *i*PASTIC provides a useful tool that enables users to render the biplot on any two arbitrary principal components.

### Testing *i*PASTIC software

To test of accuracy the software, two data sets gathered from two experiments were used to screen for the most tolerant genotypes in response to severe salinity and water deficit stresses. In Data Set 1, we tested 90 genotypes and accessions of cultivated and wild relatives of wheat under control and saline conditions. A greenhouse pot experiment was conducted in 2015–2016 at the Crop Production and Breeding Department, Imam Khomeini International University, Qazvin, Iran. Information on experimental setup, growth conditions, salinity treatments, and collection of aboveground biomass yield under control (Yp) and saline (Ys) conditions for each seedling plant is in Ahmadi et al. (2018b). Detailed information on the tested genotypes is in Appendix S1. Results of the nine yield‐based indices, along with relative change due to stress for each genotype, are shown in Appendix S2. In the control conditions, shoot dry weight (Yp) ranged from 37.65 to 99.08 mg·plant^−1^, and genotypes G1, G3, G25, G20, and G30 had the highest mean performance. Under salinity stress, shoot dry weight (Ys) ranged from 25.43 to 84.38 mg·plant^−1^, and genotypes G3, G47, G2, G20, and G46 showed the highest values. The relative change due to salinity stress for each tested genotype revealed that the genotypes G55, G47, G69, G71, and G46 had the smallest changes, being 2.19%, 2.53%, 2.86%, 4.59%, and 5.46% lower than the controls. Using the TOL index, genotypes with lower values are more tolerant to stress. Accordingly, genotypes G55, G69, G47, G71, and G23 were the most tolerant to salinity, and genotypes G88, G25, G34, G59, and G4 were the most sensitive. Genotypes that perform well under non‐stress and stressful conditions will have high values for the STI, MP, GMP, and HM indices and will be identified as tolerant. In this case, genotypes G2, G3, G20, G46, G47, and G50 had the highest values for these indices. The SSI identifies only those genotypes with minimal reductions under stressful compared to non‐stressful conditions (Fischer and Maurer, 1978); an SSI > 1 indicates above‐average susceptibility to drought stress (Guttieri et al., 2001). As shown in Appendix S2, the majority of genotypes had an SSI ≤ 1; with G55, G47, G69, G71, and G46 having the lowest values. Three indices (YI, YSI, and RSI) can be used to evaluate genotypic stability in both stressful and non‐stressful conditions. These indices are based on tolerance or susceptibility of genotypes, and have been used in many crops, including bread wheat (Sardouei‐Nasab et al., 2019), durum wheat (Etminan et al., 2019), barley (Khalili et al., 2016), safflower (Khalili et al., 2014), chickpea (Pour‐Siabidi and Pour‐Aboughadareh, 2013), and potato (Cabello et al., 2013). YSI and RSI produced similar ranking patterns in the characterization of tolerant genotypes, with G55, G47, G69, G71, and G46 having the highest values.

Identifying tolerant genotypes based on a single index could be problematic, as seen here. Our program can estimate an ASR for all indices to select potentially superior genotypes; the lower the value, the more superior the genotype. In this case, G47 (ASR = 3.73; SD = 2.90), G46 (ASR = 6.82; SD = 4.09), and G2 (ASR = 9.64; SD = 9.08) were the most salinity‐tolerant genotypes in severe salinity conditions (Appendix S3). The relative frequency results provided more information on the distribution of genotypes into different classes. For example, under control conditions, half of the genotypes had a yield potential from 51 to 65 mg·plant^−1^, but under salinity stress the yield potential of most genotypes ranged from 34 to 62 mg·plant^−1^ (Appendix S4). The relative frequencies of the genotypes based on other indices are presented in Appendix [Link], [Link], [Link]. Two heat maps based on the actual values of indices and their ranking patterns across all genotypes revealed that STI, MP, GMP, and HM are strongly correlated with crop performance (Yp and Ys) (Appendix S7). The highly significant correlations between these indices and yield under control and saline conditions indicate their capacity to identify genotypes with high potential yield and tolerance to saline conditions. Furthermore, the highly significant correlation between these indices suggests that they can be used interchangeably to select tolerant genotypes. In contrast, SSI, TOL, YSI, and YI were strongly correlated to Ys but not Yp, and therefore cannot be used to identify Group A genotypes. The ability to separate Group A genotypes from others using STI, GMP, and MP is consistent with the findings reported for common bean (Fernandez, 1992), chickpea (Ganjeali et al., 2011), and canola (Khalili et al., 2012). Appendix S8 shows rendered 3D plots based on the STI index and yield (Yp and Ys). To demonstrate the opt‐in functionality of the software, we have shown plots from different angles. Genotypes G2, G3, G8, G9, G10, G20, G43, G45, G46, G47, G48, G49, G50, G54, G61, and G83 were placed in Group A. The PCA results based on the correlation matrix indicated that the first two principal components with eigenvalues >1 accounted for 99.26% of the total variation in yield performance and nine yield‐based indices (outputs including descriptive statistics, correlation and/or covariance matrix, eigenvalues, eigenvectors, factor loading, contribution of variables in each component, and factor scores for each genotype not shown). PC1 was positively influenced by yield (Yp and Ys) and all indices except SSI and TOL, whereas PC2 was positively influenced by Yp, TOL, MP, GMP, HM, and SSI. Hence, selection based on high values of PC1 and intermediate values of PC2 could help to identify salt‐tolerant genotypes. Several genotypes, including G2, G3, G20, G46, G47, and G50, were identified as superior genotypes, which is supported by the findings in the 3D plot (Appendix S9).

In Data Set 2, we tested the software using shoot dry weight data from a water‐stress experiment that included nine genotypes from several species of cultivated and wild wheat—*Triticum* *aestivum* L., *T. durum* Desf., *T. urartu* Thumanjan ex Gandilyan, *T. boeoticum* Boiss., *Aegilops tauschii* Coss., *Ae. neglecta* Req. ex Bertol., *Ae. triuncialis* L., *Ae. crassa* Boiss., and *Ae. caudata* L. All genotypes were grown in a greenhouse maintained at an optimal photoperiod and growing temperature at the Department of Genetics and Plant Breeding, Imam Khomeini International University, Qazvin, Iran, during 2016–2017. The experiment was arranged as factorial using randomized complete block design with three replications. The plants were well watered every 1–2 days to maintain 90% ± 5% field capacity (FC). The water stress treatment (FC = 25 ± 5%) was initiated at the three‐leaf stage of growth. Details on the growing conditions, stress treatments, and data collection are available in Ahmadi et al. (2018a). The results of this data set are summarized in Appendix S10. Under both control and water‐stress conditions, *T. urartu* had the highest dry matter, followed by *T. aestivum* and *T. durum*, whereas *Ae. crassa* and *Ae. tauschii* had the lowest. However, *Ae. neglecta*,* Ae. tauschii*, and *T. durum* had the smallest changes in dry weight in response to water stress. Based on the lowest values for TOL and SSI and highest values for RSI and YSI, *Ae. neglecta* and *Ae. tauschii* were selected as the most tolerant genotypes. In contrast, *T. urartu*,* T. durum*, and *T. aestivum* had the highest values for STI, MP, GMP, HM, and YI. Based on ASR values, the most tolerant genotypes were *T. durum* (2.27), *T. aestivum* (3.64), *Ae. neglecta* (4.18), and *Ae. caudata* (4.36) (Appendix S11). The correlation coefficients for yield performance and the nine indices revealed that STI, MP, GMP, HM, and YI were strongly correlated with both Yp and Ys (Appendix S12); hence, these indices were used to generate a 3D plot to identify genotypes into Fernandezʼs groups. As shown in Appendix S13, Group A comprised *T. durum*,* T. aestivum*,* T. urartu*, and *Ae. caudata*; Group B contained *Ae. triuncialis*; Group C contained *Ae. neglecta*; and Group D comprised *Ae. tauschii*,* Ae. crassa*, and *T. boeoticum*.

Because PCA is a multivariate analysis commonly used for reducing data through decomposing the total variance into a few new independent components, achieving acceptable results will depend on the size of the data set. For this data set, we only used PCA analysis to demonstrate the applicability of the software. Here, the first two principal components accounted for 98.86% (PC1 = 73.60% and PC2 = 25.26%) of the total variation in yield performance and the measured indices. Eigenvector coefficients revealed that Yp and Ys along with the indices, except YSI and RSI, had a positive association with PC1, whereas SSI and TOL had a negative association with PC2. Hence, using the PC1 results, tolerant genotypes will be selected based on high‐ranking yield performance and tolerance indices such as MP, GMP, and STI. In this case, *T. aestivum* and *T. durum* were identified as the tolerant genotypes with acceptable performance under both non‐stress and water‐stress conditions (Appendix S14). Regarding the PCA tool, it is worth noting that while this method provides a good way of summarizing data when interesting patterns increase the variance of projections onto orthogonal components, it also has limitations worth considering when interpreting the output. First, the underlying structure of the data must be linear. Second, patterns that are highly correlated may be unresolved because all principal components are uncorrelated. Finally, the goal is to maximize variance and not necessarily to find clusters (Lever et al., 2017).

## CONCLUSIONS

We developed a novel online software (*i*PASTIC) to calculate several yield‐based stress tolerance and susceptibility indices that are important in the identification of tolerant crop genotypes. In addition to the useful and practical tools described above in the Methods and Results, *i*PASTIC also has the following advantages: (1) It can analyze large data sets in minimal time; (2) It is a cross‐platform software that does not require additional downloads or installation; (3) Unlike other codes based on SAS and R packages, which require additional user knowledge, *i*PASTIC has a web‐based user‐friendly interface; and (4) It is compatible with the major browsers (e.g., Google Chrome, Mozilla Firefox, Safari). These advantages, combined with its user‐friendly interface and tools for better selection of entry genotypes, make *i*PASTIC valuable for use in agronomy and plant breeding programs by students, teachers, and researchers alike.

## Supporting information


**APPENDIX S1.** Label, GenBank accession number, and species of the 90 wheat genotypes and accessions tested in Data Set 1.

**APPENDIX S2.** Yield performance of 90 wheat genotypes and accessions under control (Yp) and saline (Ys) conditions along with the relative change (RC) due to stress and tolerance and susceptibility indices calculated using *i*PASTIC software for Data Set 1.

**APPENDIX S3.** Yield performance rankings of 90 wheat genotypes and accessions under control (Yp) and saline (Ys) conditions along with the calculated tolerance and susceptibility indices using *i*PASTIC software for Data Set 1.

**APPENDIX S4.** (A–B) Relative frequency of yield performances under (A) control conditions and (B) stress conditions in 90 wheat genotypes and accessions. (C–D) Relative frequency of (C) mean productivity (MP) indices and (D) geometric mean productivity (GMP) indices calculated by *i*PASTIC software for Data Set 1.

**APPENDIX S5.** Relative frequency of (A) relative stress index (RSI), (B) stress tolerance index (STI), (C) stress susceptability index (SSI), and (D) yield index (YI) indices calculated by *i*PASTIC software for Data Set 1.

**APPENDIX S6.** Relative frequency of (A) yield stability index (YSI), (B) harmonic mean (HM), and (C) tolerance index (TOL) indices calculated by *i*PASTIC software for Data Set 1.

**APPENDIX S7.** Rendered heat‐map plot based on Pearsonʼs correlation analysis for Data Set 1. See Table 1 for full definitions of indices.

**APPENDIX S8.** Rendered three‐dimensional plot based on the STI index and yield performance (Yp and Ys) of the 90 wheat genotypes and accessions in Data Set 1. Each plot shows a view angle of distribution of entry genotypes into Fernandezʼs groups (A–D).

**APPENDIX S9.** Rendered principal components analysis–based biplot based on the correlation matrix of Yp, Ys, and nine tolerance and susceptibility indices calculated using *i*PASTIC software for Data Set 1.

**APPENDIX S10.** Yield performance of nine wheat genotypes under control (Yp) and saline (Ys) conditions along with the relative change (RC) due to stress and tolerance and susceptibility indices calculated using *i*PASTIC software for Data Set 2.

**APPENDIX S11.** Yield performance rankings of nine wheat genotypes under control (Yp) and saline (Ys) conditions along with the calculated tolerance and susceptibility indices using *i*PASTIC software for Data Set 2.

**APPENDIX S12.** Rendered heat‐map plot based on Pearsonʼs correlation analysis for Data Set 2. See Table 1 for full definitions of indices.

**APPENDIX 13.** Rendered three‐dimensional plot based on the STI index and yield performance (Yp and Ys) of the 90 wheat genotypes and accessions in Data Set 2.

**APPENDIX 14.** Rendered principal components analysis–based biplot based on the correlation matrix of Yp, Ys, and nine tolerance and susceptibility indices calculated using *i*PASTIC software for Data Set 2.

## Data Availability

The R script source codes used to develop *i*PASTIC, as well as the supporting data sets, are available on GitHub (https://github.com/pour-aboughadareh/iPASTIC/) and the *i*PASTIC web application is available at https://mohsenyousefian.com/ipastic/.
