# Supplementary material for: iPASTIC: An online toolkit to estimate plant abiotic stress indices
Source: Appl Plant Sci. 2019 Jul 17;7(7):e11278. doi: 10.1002/aps3.11278 (PMC6636621; doi:10.1002/aps3.11278)
Supplement: Supplementary file 1 — APPENDIX S1. Label, GenBank accession number, and species of the 90 wheat genotypes and accessions tested in Data Set 1. [file APS3-7-e11278-s001.docx]

**APPENDIX S1.** Label, GenBank accession number, and species of the 90 wheat genotypes and accessions tested in Data Set 1.^a^

| **Label** | **GenBank accession no.** | **Species** |
| --- | --- | --- |
| G1 | IUGB-00447 | *T. aestivum* |
| G2 | IUGB-00133 | *T. aestivum* |
| G3 | IUGB-00134 | *T. aestivum* |
| G4 | IUGB-00264 | *T. aestivum* |
| G5 | IUGB-00447 | *T. aestivum* |
| G6 | IUGB-00453 | *T. aestivum* |
| G7 | IUGB-00460 | *T. aestivum* |
| G8 | IUGB-00480 | *T. aestivum* |
| G9 | IUGB-00482 | *T. aestivum* |
| G10 | IUGB-00485 | *T. aestivum* |
| G11 | IUGB-00348 | *T. boeoticum* |
| G12 | IUGB-00368 | *T. boeoticum* |
| G13 | IUGB-00102 | *T. boeoticum* |
| G14 | IUGB-00122 | *T. boeoticum* |
| G15 | IUGB-00009 | *T. boeoticum* |
| G16 | IUGB-00123 | *T. boeoticum* |
| G17 | IUGB-01865 | *T. boeoticum* |
| G18 | IUGB-00126 | *T. boeoticum* |
| G19 | IUGB-00003 | *T. boeoticum* |
| G20 | IUGB-00114 | *T. boeoticum* |
| G21 | IUGB-00334 | *Ae. crassa* |
| G22 | IUGB-00817 | *Ae. crassa* |
| G23 | IUGB-00379 | *Ae. crassa* |
| G24 | IUGB-00280 | *Ae. crassa* |
| G25 | IUGB-00170 | *Ae. crassa* |
| G26 | IUGB-00319 | *Ae. crassa* |
| G27 | IUGB-01582 | *Ae. crassa* |
| G28 | IUGB-00408 | *Ae. crassa* |
| G29 | IUGB-01564 | *Ae. crassa* |
| G30 | IUGB-00830 | *Ae. crassa* |
| G31 | IUGB-00189 | *Ae. cylindrica* |
| G32 | IUGB-00189 | *Ae. cylindrica* |
| G33 | IUGB-02073 | *Ae. cylindrica* |
| G34 | IUGB-00221 | *Ae. cylindrica* |
| G35 | IUGB-00267 | *Ae. cylindrica* |
| G36 | IUGB-00168 | *Ae. cylindrica* |
| G37 | IUGB-00188 | *Ae. cylindrica* |
| G38 | IUGB-00150 | *Ae. cylindrica* |
| G39 | IUGB-00236 | *Ae. cylindrica* |
| G40 | IUGB-00185 | *Ae. cylindrica* |
| G41 | IUGB-01731 | *T. durum* |
| G42 | IUGB-00770 | *T. durum* |
| G43 | IUGB-00531 | *T. durum* |
| G44 | IUGB-00517 | *T. durum* |
| G45 | IUGB-01859 | *T. durum* |
| G46 | IUGB-00651 | *T. durum* |
| G47 | IUGB-00268 | *T. durum* |
| G48 | IUGB-01692 | *T. durum* |
| G49 | IUGB-00522 | *T. durum* |
| G50 | IUGB-00038 | *T. durum* |
| G51 | IUGB-00224 | *Ae. tauschii* |
| G52 | IUGB-00261 | *Ae. tauschii* |
| G53 | IUGB-00223 | *Ae. tauschii* |
| G54 | IUGB-00275 | *Ae. tauschii* |
| G55 | IUGB-00366 | *Ae. tauschii* |
| G56 | IUGB-00164 | *Ae. tauschii* |
| G57 | IUGB-00020 | *Ae. tauschii* |
| G58 | IUGB-00107 | *Ae. tauschii* |
| G59 | IUGB-00365 | *Ae. tauschii* |
| G60 | IUGB-00245 | *Ae. tauschii* |
| G61 | IUGB-00317 | *Ae. triuncialis* |
| G62 | IUGB-00069 | *Ae. triuncialis* |
| G63 | IUGB-00186 | *Ae. triuncialis* |
| G64 | IUGB-00242 | *Ae. triuncialis* |
| G65 | IUGB-00139 | *Ae. triuncialis* |
| G66 | IUGB-00228 | *Ae. triuncialis* |
| G67 | IUGB-00259 | *Ae. triuncialis* |
| G68 | IUGB-01146 | *Ae. triuncialis* |
| G69 | IUGB-00031 | *Ae. triuncialis* |
| G70 | IUGB-00026 | *Ae. triuncialis* |
| G71 | IUGB-01056 | *Ae. umbellulata* |
| G72 | IUGB-01547 | *Ae. umbellulata* |
| G73 | IUGB-01429 | *Ae. umbellulata* |
| G74 | IUGB-01529 | *Ae. umbellulata* |
| G75 | IUGB-01259 | *Ae. umbellulata* |
| G76 | IUGB-01429 | *Ae. umbellulata* |
| G77 | IUGB-00887 | *Ae. umbellulata* |
| G78 | IUGB-00818 | *Ae. umbellulata* |
| G79 | IUGB-00234 | *Ae. umbellulata* |
| G80 | IUGB-00237 | *Ae. umbellulata* |
| G81 | IUGB-00079 | *T. urartu* |
| G82 | IUGB-00423 | *T. urartu* |
| G83 | IUGB-00124 | *T. urartu* |
| G84 | IUGB-00206 | *T. urartu* |
| G85 | IUGB-00077 | *T. urartu* |
| G86 | IUGB-00426 | *T. urartu* |
| G87 | IUGB-00324 | *T. urartu* |
| G88 | IUGB-00094 | *T. urartu* |
| G89 | IUGB-00162 | *T. urartu* |
| G90 | IUGB-00347 | *T. urartu* |

^a^All accessions used in this study were deposited at the Department of Agronomy and Plant Breeding, Faculty of Agriculture, Ilam University, Ilam, Iran.
