# Supplementary material for: iPASTIC: An online toolkit to estimate plant abiotic stress indices
Source: Appl Plant Sci. 2019 Jul 17;7(7):e11278. doi: 10.1002/aps3.11278 (PMC6636621; doi:10.1002/aps3.11278)
Supplement: Supplementary file 2 — APPENDIX S2. Yield performance of 90 wheat genotypes and accessions under control (Yp) and saline (Ys) conditions along with the relative change (RC) due to stress and tolerance and susceptibility indices calculated using iPASTIC software for Data Set 1. [file APS3-7-e11278-s002.docx]

**APPENDIX S2.** Yield performance of 90 wheat genotypes and accessions under control (Yp) and saline (Ys) conditions along with the relative change (RC) due to stress and tolerance and susceptibility indices calculated using *i*PASTIC software for Data Set 1.^a^

| **Genotype label** | **Yp** | **Ys** | **RC** | **TOL** | **MP** | **GMP** | **HM** | **SSI** | **STI** | **YI** | **YSI** | **RSI** |
| --- | --- | --- | --- | --- | --- | --- | --- | --- | --- | --- | --- | --- |
| G1 | 66.12 | 53.51 | 19.07 | 12.61 | 59.82 | 59.48 | 59.15 | 0.72 | 0.85 | 1.13 | 0.81 | 1.10 |
| G2 | 86.85 | 75.18 | 13.44 | 11.67 | 81.02 | 80.80 | 80.59 | 0.51 | 1.57 | 1.59 | 0.87 | 1.18 |
| G3 | 98.65 | 84.38 | 14.47 | 14.27 | 91.52 | 91.24 | 90.96 | 0.54 | 2.00 | 1.78 | 0.86 | 1.17 |
| G4 | 74.23 | 35.11 | 52.70 | 39.12 | 54.67 | 51.05 | 47.67 | 1.98 | 0.63 | 0.74 | 0.47 | 0.64 |
| G5 | 63.35 | 54.70 | 13.65 | 8.65 | 59.03 | 58.87 | 58.71 | 0.51 | 0.83 | 1.15 | 0.86 | 1.18 |
| G6 | 74.43 | 49.42 | 33.60 | 25.01 | 61.93 | 60.65 | 59.40 | 1.26 | 0.88 | 1.04 | 0.66 | 0.90 |
| G7 | 63.66 | 49.17 | 22.76 | 14.49 | 56.42 | 55.95 | 55.48 | 0.86 | 0.75 | 1.04 | 0.77 | 1.05 |
| G8 | 85.61 | 59.08 | 30.99 | 26.53 | 72.35 | 71.12 | 69.91 | 1.17 | 1.21 | 1.25 | 0.69 | 0.94 |
| G9 | 86.34 | 59.89 | 30.63 | 26.45 | 73.12 | 71.91 | 70.72 | 1.15 | 1.24 | 1.26 | 0.69 | 0.94 |
| G10 | 74.34 | 56.07 | 24.58 | 18.27 | 65.21 | 64.56 | 63.93 | 0.92 | 1.00 | 1.18 | 0.75 | 1.03 |
| G11 | 43.32 | 28.16 | 35.00 | 15.16 | 35.74 | 34.93 | 34.13 | 1.32 | 0.29 | 0.59 | 0.65 | 0.89 |
| G12 | 60.46 | 43.70 | 27.72 | 16.76 | 52.08 | 51.40 | 50.73 | 1.04 | 0.63 | 0.92 | 0.72 | 0.98 |
| G13 | 54.76 | 45.54 | 16.84 | 9.22 | 50.15 | 49.94 | 49.73 | 0.63 | 0.60 | 0.96 | 0.83 | 1.13 |
| G14 | 60.88 | 37.26 | 38.80 | 23.62 | 49.07 | 47.63 | 46.23 | 1.46 | 0.54 | 0.79 | 0.61 | 0.83 |
| G15 | 57.20 | 42.90 | 25.00 | 14.30 | 50.05 | 49.54 | 49.03 | 0.94 | 0.59 | 0.91 | 0.75 | 1.02 |
| G16 | 58.79 | 54.65 | 7.04 | 4.14 | 56.72 | 56.68 | 56.64 | 0.26 | 0.77 | 1.15 | 0.93 | 1.27 |
| G17 | 54.30 | 43.15 | 20.53 | 11.15 | 48.73 | 48.41 | 48.09 | 0.77 | 0.56 | 0.91 | 0.79 | 1.08 |
| G18 | 62.29 | 58.08 | 6.76 | 4.21 | 60.19 | 60.15 | 60.11 | 0.25 | 0.87 | 1.23 | 0.93 | 1.27 |
| G19 | 62.04 | 49.29 | 20.55 | 12.75 | 55.67 | 55.30 | 54.93 | 0.77 | 0.73 | 1.04 | 0.79 | 1.08 |
| G20 | 88.51 | 74.00 | 16.39 | 14.51 | 81.26 | 80.93 | 80.61 | 0.62 | 1.57 | 1.56 | 0.84 | 1.14 |
| G21 | 78.98 | 48.15 | 39.04 | 30.83 | 63.57 | 61.67 | 59.83 | 1.47 | 0.91 | 1.02 | 0.61 | 0.83 |
| G22 | 51.82 | 35.39 | 31.71 | 16.43 | 43.61 | 42.82 | 42.06 | 1.19 | 0.44 | 0.75 | 0.68 | 0.93 |
| G23 | 45.76 | 42.10 | 8.00 | 3.66 | 43.93 | 43.89 | 43.85 | 0.30 | 0.46 | 0.89 | 0.92 | 1.25 |
| G24 | 53.22 | 33.52 | 37.02 | 19.70 | 43.37 | 42.24 | 41.13 | 1.39 | 0.43 | 0.71 | 0.63 | 0.86 |
| G25 | 90.88 | 42.08 | 53.70 | 48.80 | 66.48 | 61.84 | 57.52 | 2.02 | 0.92 | 0.89 | 0.46 | 0.63 |
| G26 | 51.04 | 32.13 | 37.05 | 18.91 | 41.59 | 40.50 | 39.44 | 1.39 | 0.39 | 0.68 | 0.63 | 0.86 |
| G27 | 51.72 | 45.63 | 11.77 | 6.09 | 48.68 | 48.58 | 48.48 | 0.44 | 0.57 | 0.96 | 0.88 | 1.20 |
| G28 | 42.66 | 34.14 | 19.97 | 8.52 | 38.40 | 38.16 | 37.93 | 0.75 | 0.35 | 0.72 | 0.80 | 1.09 |
| G29 | 60.81 | 29.75 | 51.08 | 31.06 | 45.28 | 42.53 | 39.95 | 1.92 | 0.43 | 0.63 | 0.49 | 0.67 |
| G30 | 88.35 | 50.05 | 43.35 | 38.30 | 69.20 | 66.50 | 63.90 | 1.63 | 1.06 | 1.06 | 0.57 | 0.77 |
| G31 | 55.20 | 47.03 | 14.80 | 8.17 | 51.12 | 50.95 | 50.79 | 0.56 | 0.62 | 0.99 | 0.85 | 1.16 |
| G32 | 68.46 | 53.20 | 22.29 | 15.26 | 60.83 | 60.35 | 59.87 | 0.84 | 0.87 | 1.12 | 0.78 | 1.06 |
| G33 | 56.51 | 45.59 | 19.32 | 10.92 | 51.05 | 50.76 | 50.47 | 0.73 | 0.62 | 0.96 | 0.81 | 1.10 |
| G34 | 72.70 | 26.28 | 63.85 | 46.42 | 49.49 | 43.71 | 38.60 | 2.40 | 0.46 | 0.55 | 0.36 | 0.49 |
| G35 | 61.96 | 44.84 | 27.63 | 17.12 | 53.40 | 52.71 | 52.03 | 1.04 | 0.67 | 0.95 | 0.72 | 0.99 |
| G36 | 55.70 | 43.32 | 22.23 | 12.38 | 49.51 | 49.12 | 48.74 | 0.84 | 0.58 | 0.91 | 0.78 | 1.06 |
| G37 | 57.75 | 43.01 | 25.52 | 14.74 | 50.38 | 49.84 | 49.30 | 0.96 | 0.60 | 0.91 | 0.74 | 1.01 |
| G38 | 72.31 | 35.63 | 50.73 | 36.68 | 53.97 | 50.76 | 47.74 | 1.91 | 0.62 | 0.75 | 0.49 | 0.67 |
| G39 | 62.03 | 41.56 | 33.00 | 20.47 | 51.80 | 50.77 | 49.77 | 1.24 | 0.62 | 0.88 | 0.67 | 0.91 |
| G40 | 69.91 | 53.08 | 24.07 | 16.83 | 61.50 | 60.92 | 60.34 | 0.91 | 0.89 | 1.12 | 0.76 | 1.03 |
| G41 | 58.43 | 47.98 | 17.88 | 10.45 | 53.21 | 52.95 | 52.69 | 0.67 | 0.67 | 1.01 | 0.82 | 1.12 |
| G42 | 55.98 | 48.90 | 12.65 | 7.08 | 52.44 | 52.32 | 52.20 | 0.48 | 0.66 | 1.03 | 0.87 | 1.19 |
| G43 | 77.42 | 55.97 | 27.71 | 21.45 | 66.70 | 65.83 | 64.97 | 1.04 | 1.04 | 1.18 | 0.72 | 0.98 |
| G44 | 67.42 | 54.15 | 19.68 | 13.27 | 60.79 | 60.42 | 60.06 | 0.74 | 0.88 | 1.14 | 0.80 | 1.09 |
| G45 | 77.62 | 61.61 | 20.63 | 16.01 | 69.62 | 69.15 | 68.69 | 0.78 | 1.15 | 1.30 | 0.79 | 1.08 |
| G46 | 75.23 | 71.12 | 5.46 | 4.11 | 73.18 | 73.15 | 73.12 | 0.21 | 1.28 | 1.50 | 0.95 | 1.29 |
| G47 | 79.13 | 77.13 | 2.53 | 2.00 | 78.13 | 78.12 | 78.12 | 0.10 | 1.47 | 1.63 | 0.97 | 1.33 |
| G48 | 76.53 | 59.77 | 21.90 | 16.76 | 68.15 | 67.63 | 67.12 | 0.82 | 1.10 | 1.26 | 0.78 | 1.06 |
| G49 | 77.80 | 55.71 | 28.39 | 22.09 | 66.76 | 65.83 | 64.93 | 1.07 | 1.04 | 1.18 | 0.72 | 0.98 |
| G50 | 83.28 | 65.80 | 20.99 | 17.48 | 74.54 | 74.03 | 73.52 | 0.79 | 1.32 | 1.39 | 0.79 | 1.08 |
| G51 | 61.66 | 40.22 | 34.77 | 21.44 | 50.94 | 49.80 | 48.68 | 1.31 | 0.60 | 0.85 | 0.65 | 0.89 |
| G52 | 58.52 | 45.56 | 22.15 | 12.96 | 52.04 | 51.63 | 51.23 | 0.83 | 0.64 | 0.96 | 0.78 | 1.06 |
| G53 | 69.44 | 54.03 | 22.19 | 15.41 | 61.74 | 61.25 | 60.77 | 0.83 | 0.90 | 1.14 | 0.78 | 1.06 |
| G54 | 66.32 | 55.73 | 15.97 | 10.59 | 61.03 | 60.79 | 60.57 | 0.60 | 0.89 | 1.18 | 0.84 | 1.14 |
| G55 | 51.65 | 50.52 | 2.19 | 1.13 | 51.09 | 51.08 | 51.08 | 0.08 | 0.63 | 1.07 | 0.98 | 1.33 |
| G56 | 60.55 | 35.60 | 41.21 | 24.95 | 48.08 | 46.43 | 44.84 | 1.55 | 0.52 | 0.75 | 0.59 | 0.80 |
| G57 | 64.12 | 46.85 | 26.93 | 17.27 | 55.49 | 54.81 | 54.14 | 1.01 | 0.72 | 0.99 | 0.73 | 1.00 |
| G58 | 55.12 | 50.31 | 8.73 | 4.81 | 52.72 | 52.66 | 52.61 | 0.33 | 0.67 | 1.06 | 0.91 | 1.24 |
| G59 | 85.98 | 41.22 | 52.06 | 44.76 | 63.60 | 59.53 | 55.72 | 1.96 | 0.85 | 0.87 | 0.48 | 0.65 |
| G60 | 55.06 | 36.03 | 34.56 | 19.03 | 45.55 | 44.54 | 43.56 | 1.30 | 0.48 | 0.76 | 0.65 | 0.89 |
| G61 | 81.72 | 56.26 | 31.16 | 25.46 | 68.99 | 67.81 | 66.64 | 1.17 | 1.10 | 1.19 | 0.69 | 0.94 |
| G62 | 61.58 | 52.85 | 14.18 | 8.73 | 57.22 | 57.05 | 56.88 | 0.53 | 0.78 | 1.12 | 0.86 | 1.17 |
| G63 | 63.23 | 49.69 | 21.41 | 13.54 | 56.46 | 56.05 | 55.65 | 0.81 | 0.75 | 1.05 | 0.79 | 1.07 |
| G64 | 59.77 | 56.07 | 6.19 | 3.70 | 57.92 | 57.89 | 57.86 | 0.23 | 0.80 | 1.18 | 0.94 | 1.28 |
| G65 | 73.23 | 54.83 | 25.13 | 18.40 | 64.03 | 63.37 | 62.71 | 0.95 | 0.96 | 1.16 | 0.75 | 1.02 |
| G66 | 67.80 | 43.99 | 35.12 | 23.81 | 55.90 | 54.61 | 53.36 | 1.32 | 0.72 | 0.93 | 0.65 | 0.88 |
| G67 | 66.59 | 49.13 | 26.22 | 17.46 | 57.86 | 57.20 | 56.54 | 0.99 | 0.79 | 1.04 | 0.74 | 1.00 |
| G68 | 74.55 | 51.53 | 30.88 | 23.02 | 63.04 | 61.98 | 60.94 | 1.16 | 0.92 | 1.09 | 0.69 | 0.94 |
| G69 | 58.01 | 56.35 | 2.86 | 1.66 | 57.18 | 57.17 | 57.17 | 0.11 | 0.78 | 1.19 | 0.97 | 1.32 |
| G70 | 57.23 | 30.73 | 46.30 | 26.50 | 43.98 | 41.94 | 39.99 | 1.74 | 0.42 | 0.65 | 0.54 | 0.73 |
| G71 | 52.07 | 49.68 | 4.59 | 2.39 | 50.88 | 50.86 | 50.85 | 0.17 | 0.62 | 1.05 | 0.95 | 1.30 |
| G72 | 46.97 | 41.11 | 12.48 | 5.86 | 44.04 | 43.94 | 43.85 | 0.47 | 0.46 | 0.87 | 0.88 | 1.19 |
| G73 | 62.65 | 39.03 | 37.70 | 23.62 | 50.84 | 49.45 | 48.10 | 1.42 | 0.59 | 0.82 | 0.62 | 0.85 |
| G74 | 54.65 | 48.93 | 10.47 | 5.72 | 51.79 | 51.71 | 51.63 | 0.39 | 0.64 | 1.03 | 0.90 | 1.22 |
| G75 | 51.69 | 36.43 | 29.52 | 15.26 | 44.06 | 43.39 | 42.74 | 1.11 | 0.45 | 0.77 | 0.70 | 0.96 |
| G76 | 57.99 | 31.59 | 45.53 | 26.40 | 44.79 | 42.80 | 40.90 | 1.71 | 0.44 | 0.67 | 0.54 | 0.74 |
| G77 | 54.36 | 40.02 | 26.38 | 14.34 | 47.19 | 46.64 | 46.10 | 0.99 | 0.52 | 0.84 | 0.74 | 1.00 |
| G78 | 42.77 | 25.43 | 40.54 | 17.34 | 34.10 | 32.98 | 31.90 | 1.53 | 0.26 | 0.54 | 0.59 | 0.81 |
| G79 | 53.03 | 32.72 | 38.30 | 20.31 | 42.88 | 41.66 | 40.47 | 1.44 | 0.42 | 0.69 | 0.62 | 0.84 |
| G80 | 50.24 | 44.14 | 12.14 | 6.10 | 47.19 | 47.09 | 46.99 | 0.46 | 0.53 | 0.93 | 0.88 | 1.20 |
| G81 | 37.65 | 31.13 | 17.32 | 6.52 | 34.39 | 34.24 | 34.08 | 0.65 | 0.28 | 0.66 | 0.83 | 1.13 |
| G82 | 53.15 | 39.33 | 26.00 | 13.82 | 46.24 | 45.72 | 45.21 | 0.98 | 0.50 | 0.83 | 0.74 | 1.01 |
| G83 | 75.95 | 57.50 | 24.29 | 18.45 | 66.73 | 66.08 | 65.45 | 0.91 | 1.05 | 1.21 | 0.76 | 1.03 |
| G84 | 62.28 | 50.11 | 19.54 | 12.17 | 56.20 | 55.86 | 55.54 | 0.74 | 0.75 | 1.06 | 0.80 | 1.10 |
| G85 | 70.50 | 40.42 | 42.67 | 30.08 | 55.46 | 53.38 | 51.38 | 1.61 | 0.68 | 0.85 | 0.57 | 0.78 |
| G86 | 61.00 | 53.15 | 12.87 | 7.85 | 57.08 | 56.94 | 56.81 | 0.48 | 0.78 | 1.12 | 0.87 | 1.19 |
| G87 | 54.81 | 35.77 | 34.74 | 19.04 | 45.29 | 44.28 | 43.29 | 1.31 | 0.47 | 0.75 | 0.65 | 0.89 |
| G88 | 99.08 | 28.87 | 70.86 | 70.21 | 63.98 | 53.48 | 44.71 | 2.67 | 0.69 | 0.61 | 0.29 | 0.40 |
| G89 | 56.64 | 50.93 | 10.08 | 5.71 | 53.79 | 53.71 | 53.63 | 0.38 | 0.69 | 1.07 | 0.90 | 1.22 |
| G90 | 66.32 | 53.03 | 20.04 | 13.29 | 59.68 | 59.30 | 58.94 | 0.75 | 0.84 | 1.12 | 0.80 | 1.09 |

^a^ See Table 1 for definitions of indices.
