# Supplementary material for: iPASTIC: An online toolkit to estimate plant abiotic stress indices
Source: Appl Plant Sci. 2019 Jul 17;7(7):e11278. doi: 10.1002/aps3.11278 (PMC6636621; doi:10.1002/aps3.11278)
Supplement: Supplementary file 3 — APPENDIX S3. Yield performance rankings of 90 wheat genotypes and accessions under control (Yp) and saline (Ys) conditions along with the calculated tolerance and susceptibility indices using iPASTIC software for Data Set 1. [file APS3-7-e11278-s003.docx]

**APPENDIX S3.** Yield performance rankings of 90 wheat genotypes and accessions under control (Yp) and saline (Ys) conditions along with the calculated tolerance and susceptibility indices using *i*PASTIC software for Data Set 1.^a^

| **Genotype label** | **Yp** | **Ys** | **TOL** | **MP** | **GMP** | **HM** | **SSI** | **STI** | **YI** | **YSI** | **RSI** | **SR** | **AR** | **SD** |
| --- | --- | --- | --- | --- | --- | --- | --- | --- | --- | --- | --- | --- | --- | --- |
| G1 | 36 | 25 | 31 | 30 | 29 | 27 | 28 | 29 | 25 | 28 | 63 | 351 | 31.91 | 10.75 |
| G2 | 6 | 3 | 28 | 3 | 3 | 3 | 18 | 3 | 3 | 18 | 73 | 161 | 14.64 | 21.20 |
| G3 | 2 | 1 | 38 | 1 | 1 | 1 | 21 | 1 | 1 | 21 | 70 | 158 | 14.36 | 22.29 |
| G4 | 23 | 78 | 86 | 46 | 55 | 67 | 87 | 55 | 78 | 87 | 4 | 666 | 60.55 | 27.53 |
| G5 | 39 | 21 | 21 | 32 | 31 | 29 | 19 | 31 | 21 | 19 | 72 | 335 | 30.45 | 15.28 |
| G6 | 21 | 39 | 75 | 23 | 24 | 26 | 66 | 24 | 39 | 66 | 25 | 428 | 38.91 | 20.37 |
| G7 | 38 | 41 | 41 | 40 | 39 | 40 | 44 | 39 | 41 | 44 | 47 | 454 | 41.27 | 2.69 |
| G8 | 9 | 10 | 80 | 8 | 8 | 8 | 62 | 8 | 10 | 62 | 29 | 294 | 26.73 | 27.58 |
| G9 | 7 | 8 | 78 | 7 | 7 | 7 | 60 | 7 | 8 | 60 | 31 | 280 | 25.45 | 27.37 |
| G10 | 22 | 15 | 58 | 17 | 16 | 15 | 47 | 16 | 15 | 47 | 44 | 312 | 28.36 | 16.82 |
| G11 | 87 | 88 | 44 | 88 | 88 | 88 | 70 | 88 | 88 | 70 | 21 | 820 | 74.55 | 22.54 |
| G12 | 53 | 56 | 50 | 53 | 53 | 55 | 57 | 53 | 56 | 57 | 34 | 577 | 52.45 | 6.49 |
| G13 | 71 | 52 | 23 | 64 | 61 | 58 | 25 | 61 | 52 | 25 | 66 | 558 | 50.73 | 17.83 |
| G14 | 50 | 71 | 72 | 68 | 69 | 69 | 76 | 69 | 71 | 76 | 15 | 706 | 64.18 | 17.71 |
| G15 | 62 | 60 | 39 | 65 | 64 | 60 | 48 | 64 | 60 | 48 | 43 | 613 | 55.73 | 9.37 |
| G16 | 55 | 22 | 8 | 38 | 37 | 35 | 8 | 37 | 22 | 8 | 83 | 353 | 32.09 | 22.70 |
| G17 | 74 | 58 | 27 | 69 | 68 | 65 | 34 | 68 | 58 | 34 | 57 | 612 | 55.64 | 16.37 |
| G18 | 42 | 11 | 9 | 29 | 27 | 22 | 7 | 27 | 11 | 7 | 84 | 276 | 25.09 | 22.58 |
| G19 | 44 | 40 | 32 | 43 | 41 | 41 | 35 | 41 | 40 | 35 | 56 | 448 | 40.73 | 6.26 |
| G20 | 4 | 4 | 42 | 2 | 2 | 2 | 24 | 2 | 4 | 24 | 67 | 177 | 16.09 | 21.50 |
| G21 | 13 | 45 | 82 | 21 | 20 | 25 | 77 | 20 | 45 | 77 | 14 | 439 | 39.91 | 27.10 |
| G22 | 79 | 77 | 49 | 83 | 80 | 79 | 64 | 80 | 77 | 64 | 27 | 759 | 69.00 | 17.26 |
| G23 | 86 | 61 | 5 | 82 | 77 | 74 | 9 | 77 | 61 | 9 | 82 | 623 | 56.64 | 32.44 |
| G24 | 75 | 80 | 64 | 84 | 83 | 80 | 72 | 83 | 80 | 72 | 19 | 792 | 72.00 | 18.60 |
| G25 | 3 | 62 | 89 | 16 | 19 | 31 | 88 | 19 | 62 | 88 | 3 | 480 | 43.64 | 34.80 |
| G26 | 83 | 82 | 61 | 86 | 86 | 85 | 73 | 86 | 82 | 73 | 18 | 815 | 74.09 | 20.17 |
| G27 | 80 | 49 | 14 | 70 | 67 | 63 | 13 | 67 | 49 | 13 | 78 | 563 | 51.18 | 26.18 |
| G28 | 89 | 79 | 20 | 87 | 87 | 87 | 32 | 87 | 79 | 32 | 59 | 738 | 67.09 | 26.64 |
| G29 | 51 | 86 | 83 | 77 | 82 | 84 | 85 | 82 | 86 | 85 | 6 | 807 | 73.36 | 24.49 |
| G30 | 5 | 36 | 85 | 10 | 12 | 16 | 81 | 12 | 36 | 81 | 10 | 384 | 34.91 | 32.10 |
| G31 | 67 | 47 | 19 | 57 | 56 | 54 | 22 | 56 | 47 | 22 | 69 | 516 | 46.91 | 17.97 |
| G32 | 30 | 26 | 45 | 27 | 26 | 24 | 43 | 26 | 26 | 43 | 48 | 364 | 33.09 | 9.44 |
| G33 | 64 | 50 | 26 | 59 | 60 | 56 | 29 | 60 | 50 | 29 | 62 | 545 | 49.55 | 14.54 |
| G34 | 25 | 89 | 88 | 67 | 78 | 86 | 89 | 78 | 89 | 89 | 2 | 780 | 70.91 | 29.67 |
| G35 | 46 | 53 | 53 | 49 | 48 | 48 | 55 | 48 | 53 | 55 | 36 | 544 | 49.45 | 5.47 |
| G36 | 66 | 57 | 30 | 66 | 66 | 61 | 42 | 66 | 57 | 42 | 49 | 602 | 54.73 | 12.35 |
| G37 | 60 | 59 | 43 | 63 | 62 | 59 | 50 | 62 | 59 | 50 | 41 | 608 | 55.27 | 7.90 |
| G38 | 26 | 75 | 84 | 47 | 59 | 66 | 84 | 59 | 75 | 84 | 7 | 666 | 60.55 | 25.19 |
| G39 | 45 | 63 | 66 | 55 | 58 | 57 | 65 | 58 | 63 | 65 | 26 | 621 | 56.45 | 11.78 |
| G40 | 28 | 28 | 52 | 25 | 22 | 21 | 45 | 22 | 28 | 45 | 46 | 362 | 32.91 | 11.59 |
| G41 | 57 | 46 | 24 | 50 | 47 | 45 | 27 | 47 | 46 | 27 | 64 | 480 | 43.64 | 12.68 |
| G42 | 65 | 44 | 17 | 52 | 50 | 47 | 16 | 50 | 44 | 16 | 75 | 476 | 43.27 | 19.59 |
| G43 | 16 | 17 | 68 | 15 | 15 | 13 | 56 | 15 | 17 | 56 | 35 | 323 | 29.36 | 20.77 |
| G44 | 32 | 23 | 34 | 28 | 25 | 23 | 31 | 25 | 23 | 31 | 60 | 335 | 30.45 | 10.59 |
| G45 | 15 | 7 | 48 | 9 | 9 | 9 | 36 | 9 | 7 | 36 | 55 | 240 | 21.82 | 18.25 |
| G46 | 19 | 5 | 7 | 6 | 6 | 6 | 5 | 6 | 5 | 5 | 86 | 156 | 14.18 | 24.16 |
| G47 | 12 | 2 | 3 | 4 | 4 | 4 | 2 | 4 | 2 | 2 | 89 | 128 | 11.64 | 25.82 |
| G48 | 17 | 9 | 50 | 12 | 11 | 10 | 39 | 11 | 9 | 39 | 52 | 259 | 23.55 | 17.56 |
| G49 | 14 | 19 | 69 | 13 | 14 | 14 | 58 | 14 | 19 | 58 | 33 | 325 | 29.55 | 21.57 |
| G50 | 10 | 6 | 57 | 5 | 5 | 5 | 37 | 5 | 6 | 37 | 54 | 227 | 20.64 | 21.19 |
| G51 | 47 | 67 | 67 | 60 | 63 | 62 | 69 | 63 | 67 | 69 | 22 | 656 | 59.64 | 13.94 |
| G52 | 56 | 51 | 33 | 54 | 52 | 51 | 40 | 52 | 51 | 40 | 51 | 531 | 48.27 | 7.21 |
| G53 | 29 | 24 | 47 | 24 | 21 | 19 | 41 | 21 | 24 | 41 | 50 | 341 | 31.00 | 11.45 |
| G54 | 34 | 18 | 25 | 26 | 23 | 20 | 23 | 23 | 18 | 23 | 68 | 301 | 27.36 | 14.17 |
| G55 | 82 | 33 | 1 | 58 | 54 | 52 | 1 | 54 | 33 | 1 | 90 | 459 | 41.73 | 31.23 |
| G56 | 52 | 76 | 74 | 71 | 72 | 72 | 79 | 72 | 76 | 79 | 12 | 735 | 66.82 | 19.59 |
| G57 | 37 | 48 | 54 | 44 | 42 | 42 | 54 | 42 | 48 | 54 | 37 | 502 | 45.64 | 6.42 |
| G58 | 68 | 34 | 10 | 51 | 49 | 46 | 10 | 49 | 34 | 10 | 81 | 442 | 40.18 | 23.57 |
| G59 | 8 | 64 | 87 | 20 | 28 | 37 | 86 | 28 | 64 | 86 | 5 | 513 | 46.64 | 31.71 |
| G60 | 69 | 73 | 62 | 75 | 74 | 76 | 67 | 74 | 73 | 67 | 24 | 734 | 66.73 | 14.81 |
| G61 | 11 | 14 | 76 | 11 | 10 | 11 | 63 | 10 | 14 | 63 | 28 | 311 | 28.27 | 25.81 |
| G62 | 48 | 30 | 22 | 35 | 35 | 33 | 20 | 35 | 30 | 20 | 71 | 379 | 34.45 | 14.61 |
| G63 | 40 | 37 | 36 | 39 | 38 | 38 | 38 | 38 | 37 | 38 | 53 | 432 | 39.27 | 4.67 |
| G64 | 54 | 15 | 6 | 33 | 32 | 30 | 6 | 32 | 15 | 6 | 85 | 314 | 28.55 | 24.03 |
| G65 | 24 | 20 | 59 | 18 | 17 | 17 | 49 | 17 | 20 | 49 | 42 | 332 | 30.18 | 16.10 |
| G66 | 31 | 55 | 73 | 42 | 43 | 44 | 71 | 43 | 55 | 71 | 20 | 548 | 49.82 | 17.09 |
| G67 | 33 | 42 | 56 | 34 | 33 | 36 | 52 | 33 | 42 | 52 | 39 | 452 | 41.09 | 8.60 |
| G68 | 20 | 31 | 70 | 22 | 18 | 18 | 61 | 18 | 31 | 61 | 30 | 380 | 34.55 | 19.73 |
| G69 | 58 | 13 | 2 | 36 | 34 | 32 | 3 | 34 | 13 | 3 | 88 | 316 | 28.73 | 26.50 |
| G70 | 61 | 85 | 79 | 81 | 84 | 83 | 83 | 84 | 85 | 83 | 8 | 816 | 74.18 | 22.99 |
| G71 | 78 | 38 | 4 | 61 | 57 | 53 | 4 | 57 | 38 | 4 | 87 | 481 | 43.73 | 29.30 |
| G72 | 85 | 65 | 13 | 80 | 76 | 75 | 15 | 76 | 65 | 15 | 76 | 641 | 58.27 | 28.80 |
| G73 | 41 | 70 | 71 | 62 | 65 | 64 | 74 | 65 | 70 | 74 | 17 | 673 | 61.18 | 17.26 |
| G74 | 72 | 43 | 12 | 56 | 51 | 49 | 12 | 51 | 43 | 12 | 79 | 480 | 43.64 | 23.13 |
| G75 | 81 | 72 | 46 | 79 | 79 | 78 | 59 | 79 | 72 | 59 | 32 | 736 | 66.91 | 16.10 |
| G76 | 59 | 83 | 77 | 78 | 81 | 81 | 82 | 81 | 83 | 82 | 9 | 796 | 72.36 | 22.10 |
| G77 | 73 | 68 | 40 | 72 | 71 | 70 | 53 | 71 | 68 | 53 | 38 | 677 | 61.55 | 13.19 |
| G78 | 88 | 90 | 55 | 90 | 90 | 90 | 78 | 90 | 90 | 78 | 13 | 852 | 77.45 | 23.91 |
| G79 | 77 | 81 | 65 | 85 | 85 | 82 | 75 | 85 | 81 | 75 | 16 | 807 | 73.36 | 19.94 |
| G80 | 84 | 54 | 15 | 72 | 70 | 68 | 14 | 70 | 54 | 14 | 77 | 592 | 53.82 | 26.80 |
| G81 | 90 | 84 | 16 | 89 | 89 | 89 | 26 | 89 | 84 | 26 | 65 | 747 | 67.91 | 30.00 |
| G82 | 76 | 69 | 37 | 74 | 73 | 71 | 51 | 73 | 69 | 51 | 40 | 684 | 62.18 | 14.53 |
| G83 | 18 | 12 | 60 | 14 | 13 | 12 | 46 | 13 | 12 | 46 | 45 | 291 | 26.45 | 18.57 |
| G84 | 43 | 35 | 29 | 41 | 40 | 39 | 30 | 40 | 35 | 30 | 61 | 423 | 38.45 | 8.93 |
| G85 | 27 | 66 | 81 | 45 | 46 | 50 | 80 | 46 | 66 | 80 | 11 | 598 | 54.36 | 22.74 |
| G86 | 49 | 27 | 18 | 37 | 36 | 34 | 17 | 36 | 27 | 17 | 74 | 372 | 33.82 | 16.65 |
| G87 | 70 | 74 | 63 | 76 | 75 | 77 | 68 | 75 | 74 | 68 | 23 | 743 | 67.55 | 15.38 |
| G88 | 1 | 87 | 90 | 19 | 45 | 73 | 90 | 45 | 87 | 90 | 1 | 628 | 57.09 | 36.51 |
| G89 | 63 | 32 | 11 | 48 | 44 | 43 | 11 | 44 | 32 | 11 | 80 | 419 | 38.09 | 22.01 |
| G90 | 34 | 29 | 35 | 31 | 30 | 28 | 33 | 30 | 29 | 33 | 58 | 370 | 33.64 | 8.39 |

*Note:* ASR = average of sum of ranks; SD = standard deviation of ranks; SR = sum of ranks; Y = yield.

^a^ See Table 1 for definitions of indices.
