# Supplementary material for: iPASTIC: An online toolkit to estimate plant abiotic stress indices
Source: Appl Plant Sci. 2019 Jul 17;7(7):e11278. doi: 10.1002/aps3.11278 (PMC6636621; doi:10.1002/aps3.11278)

APPENDIX S4. (A–B) Relative frequency of yield performances under (A) control conditions and (B) stress conditions in 90 wheat genotypes and accessions. (C–D) Relative frequency of (C) mean productivity (MP) indices and (D) geometric mean productivity (GMP) indices calculated by iPASTIC software for Data Set 1.

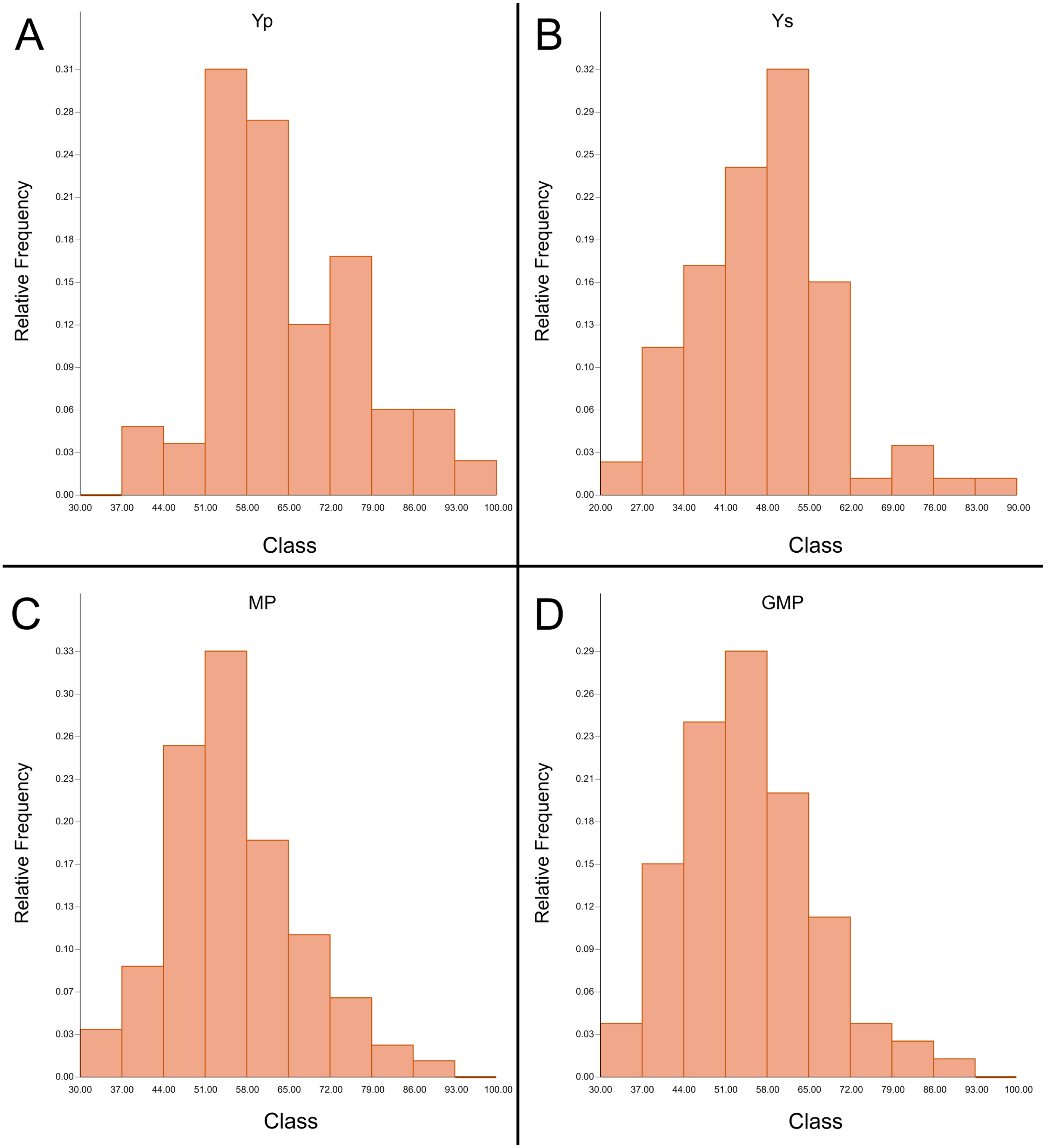

Supplement: Supplementary file 4 — APPENDIX S4. (A–B) Relative frequency of yield performances under (A) control conditions and (B) stress conditions in 90 wheat genotypes and accessions. (C–D) Relative frequency of (C) mean productivity (MP) indices and (D) geometric mean productivity (GMP) indices calculated by iPASTIC software for Data Set 1. [file APS3-7-e11278-s004.pdf]
