# Supplementary material for: iPASTIC: An online toolkit to estimate plant abiotic stress indices
Source: Appl Plant Sci. 2019 Jul 17;7(7):e11278. doi: 10.1002/aps3.11278 (PMC6636621; doi:10.1002/aps3.11278)

APPENDIX S5. Relative frequency of (A) relative stress index (RSI), (B) stress tolerance index (STI), (C) stress susceptability index (SSI), and (D) yield index (YI) indices calculated by *i*PASTIC software for Data Set 1.

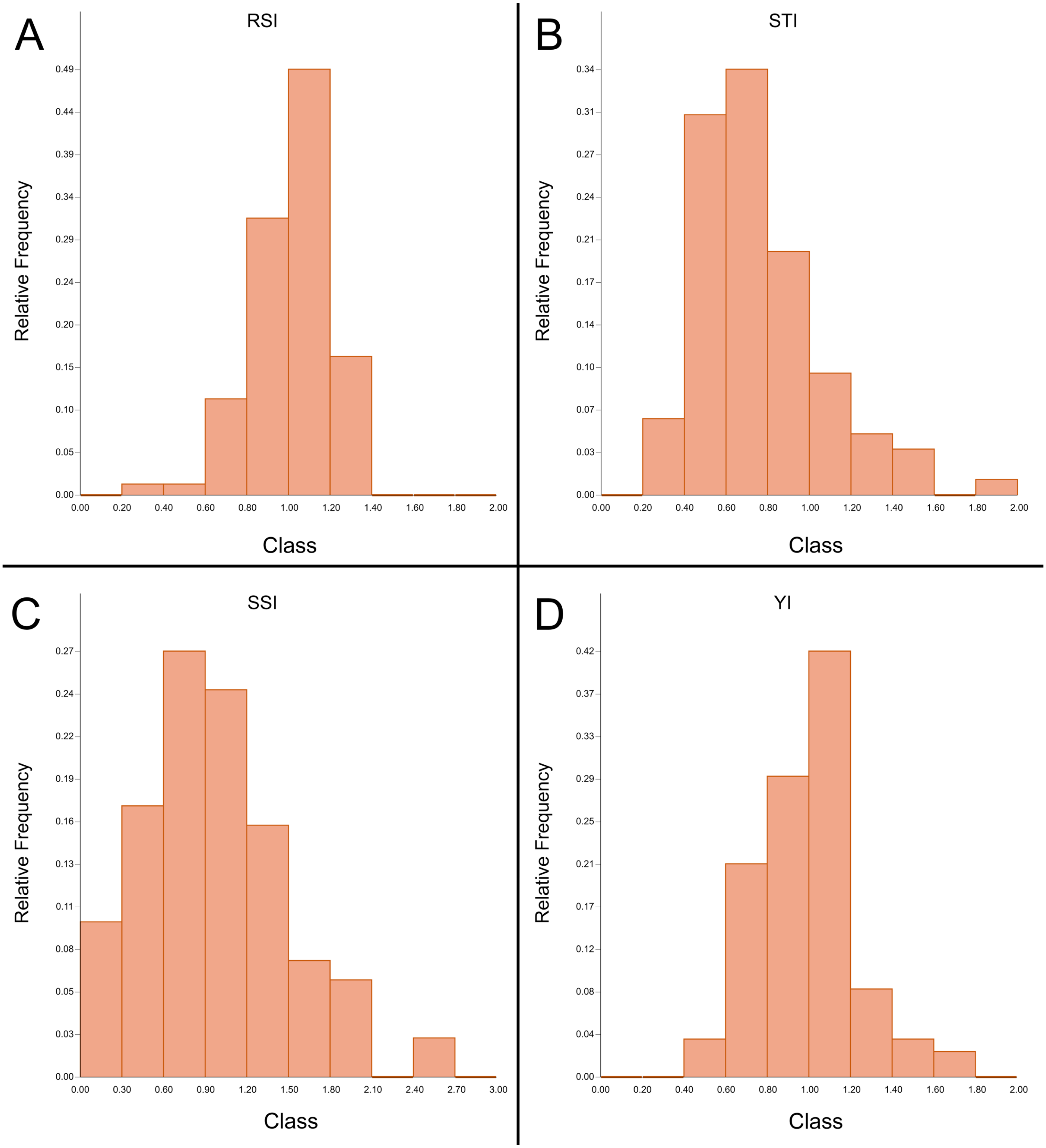

Supplement: Supplementary file 5 — APPENDIX S5. Relative frequency of (A) relative stress index (RSI), (B) stress tolerance index (STI), (C) stress susceptability index (SSI), and (D) yield index (YI) indices calculated by iPASTIC software for Data Set 1. [file APS3-7-e11278-s005.pdf]
