# Supplementary material for: iPASTIC: An online toolkit to estimate plant abiotic stress indices
Source: Appl Plant Sci. 2019 Jul 17;7(7):e11278. doi: 10.1002/aps3.11278 (PMC6636621; doi:10.1002/aps3.11278)

Pour-Aboughadareh et al.—Applications in Plant Sciences 2019 7(7)—Data Supplement S6
DOI

APPENDIX S6. Relative frequency of (A) yield stability index (YSI), (B) harmonic mean (HM), and (C) tolerance index (TOL) indices calculated by *i*PASTIC software for Data Set 1.

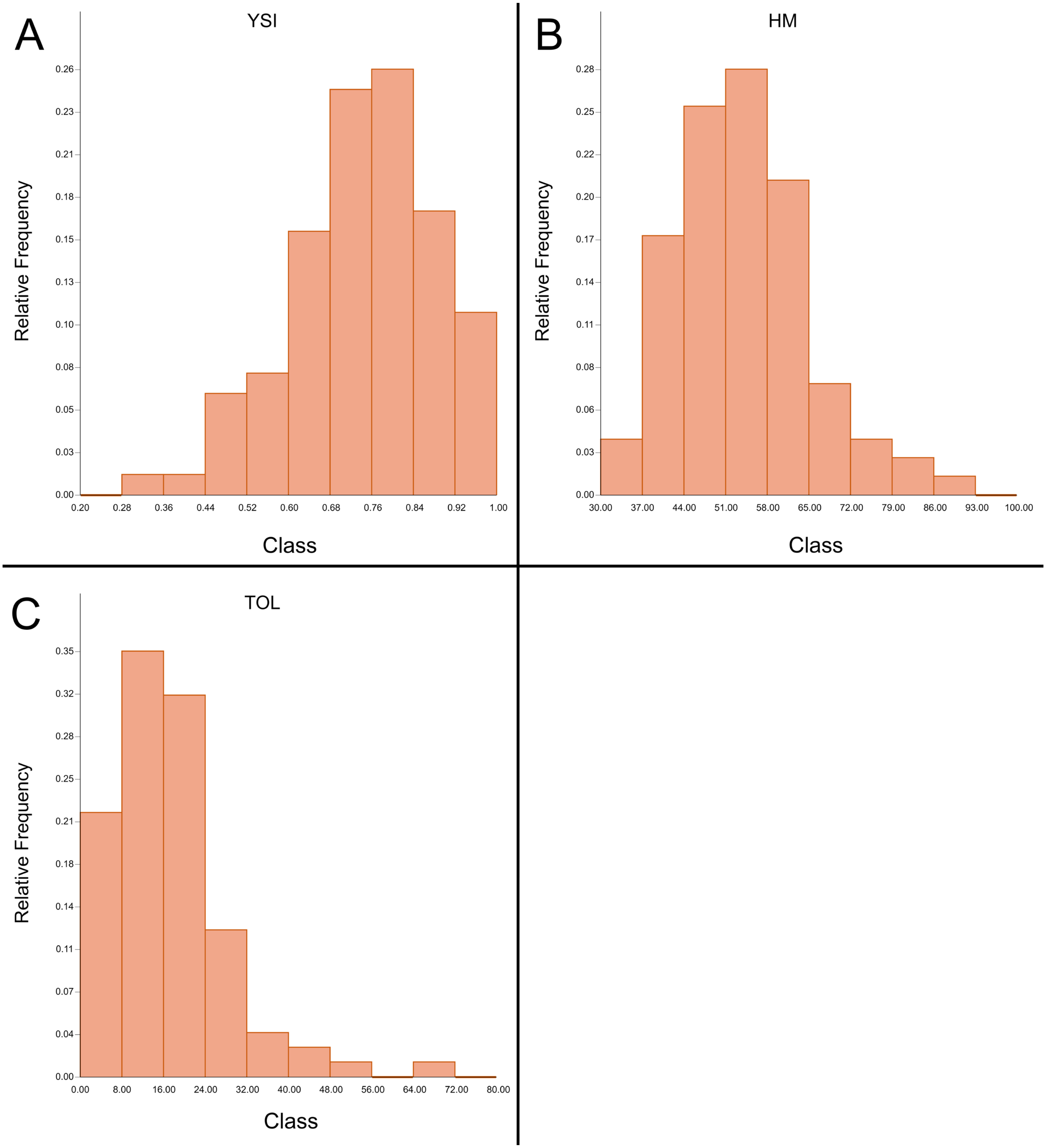

Supplement: Supplementary file 6 — APPENDIX S6. Relative frequency of (A) yield stability index (YSI), (B) harmonic mean (HM), and (C) tolerance index (TOL) indices calculated by iPASTIC software for Data Set 1. [file APS3-7-e11278-s006.pdf]
