# Supplementary material for: iPASTIC: An online toolkit to estimate plant abiotic stress indices
Source: Appl Plant Sci. 2019 Jul 17;7(7):e11278. doi: 10.1002/aps3.11278 (PMC6636621; doi:10.1002/aps3.11278)

APPENDIX S7. Rendered heat-map plot based on Pearson's correlation analysis for Data Set 1. See Table 1 for full definitions of indices.

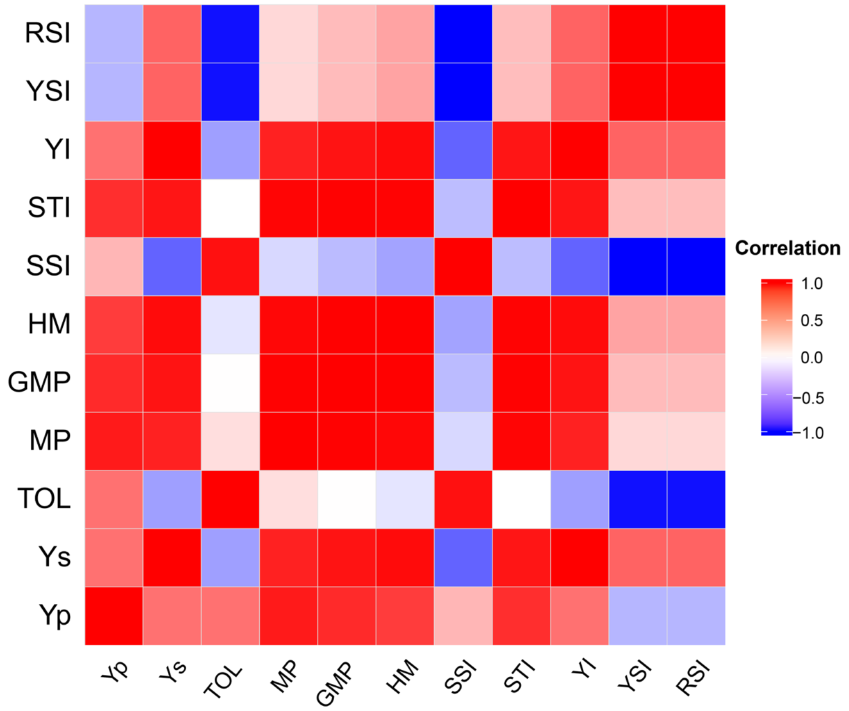

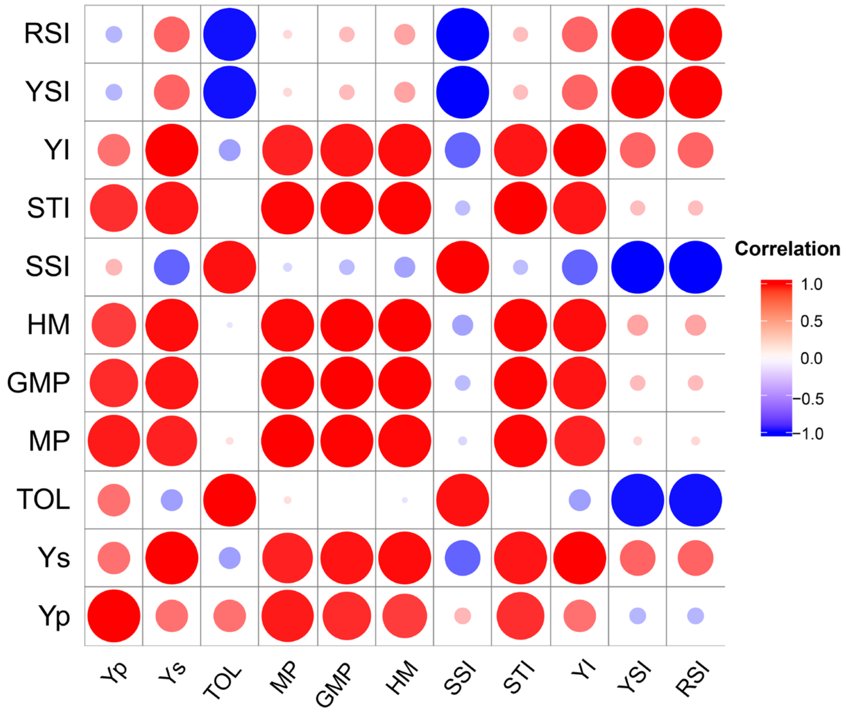

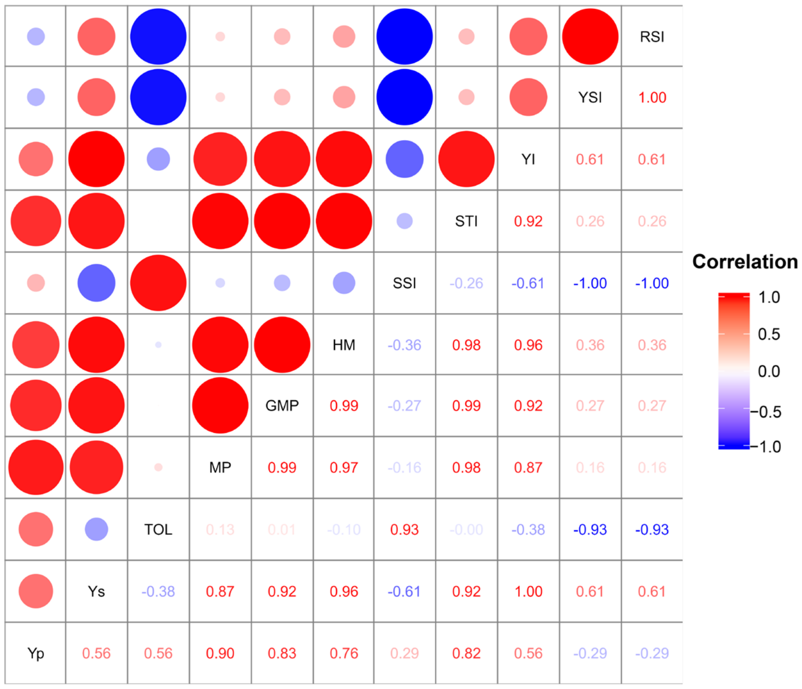

Supplement: Supplementary file 7 — APPENDIX S7. Rendered heat‐map plot based on Pearsonʼs correlation analysis for Data Set 1. See Table 1 for full definitions of indices. [file APS3-7-e11278-s007.pdf]
