# Supplementary material for: iPASTIC: An online toolkit to estimate plant abiotic stress indices
Source: Appl Plant Sci. 2019 Jul 17;7(7):e11278. doi: 10.1002/aps3.11278 (PMC6636621; doi:10.1002/aps3.11278)

APPENDIX S8. Rendered three-dimensional plot based on the STI index and yield performance (Yp and Ys) of the 90 wheat genotypes and accessions in Data Set 1. Each plot shows a view angle of distribution of entry genotypes into Fernandez's groups (A–D).

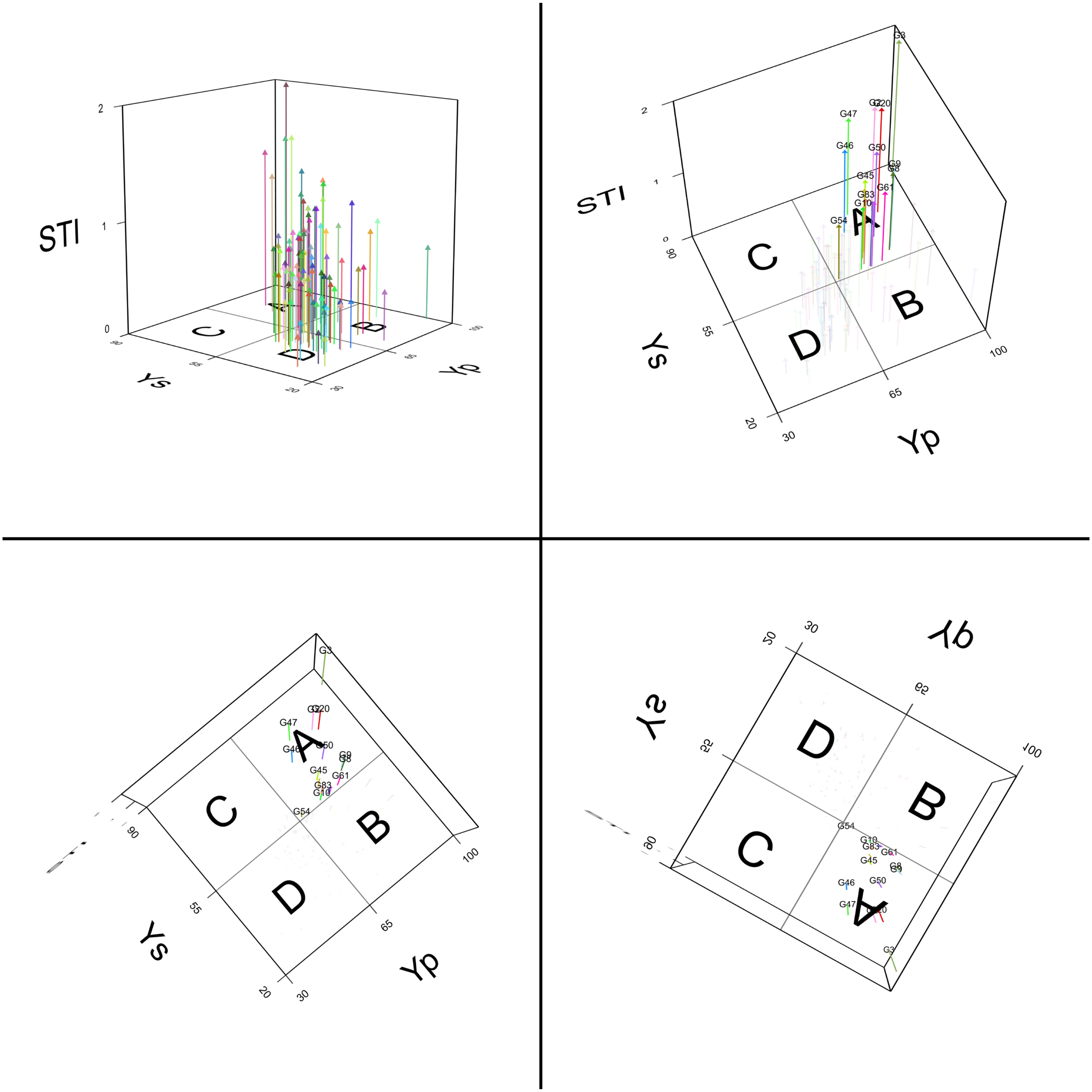

Supplement: Supplementary file 8 — APPENDIX S8. Rendered three‐dimensional plot based on the STI index and yield performance (Yp and Ys) of the 90 wheat genotypes and accessions in Data Set 1. Each plot shows a view angle of distribution of entry genotypes into Fernandezʼs groups (A–D). [file APS3-7-e11278-s008.pdf]
