# Supplementary material for: iPASTIC: An online toolkit to estimate plant abiotic stress indices
Source: Appl Plant Sci. 2019 Jul 17;7(7):e11278. doi: 10.1002/aps3.11278 (PMC6636621; doi:10.1002/aps3.11278)

DOI

APPENDIX S9. Rendered principal components analysis–based biplot based on the correlation matrix of Yp, Ys, and nine tolerance and susceptibility indices calculated using *i*PASTIC software for Data Set 1.

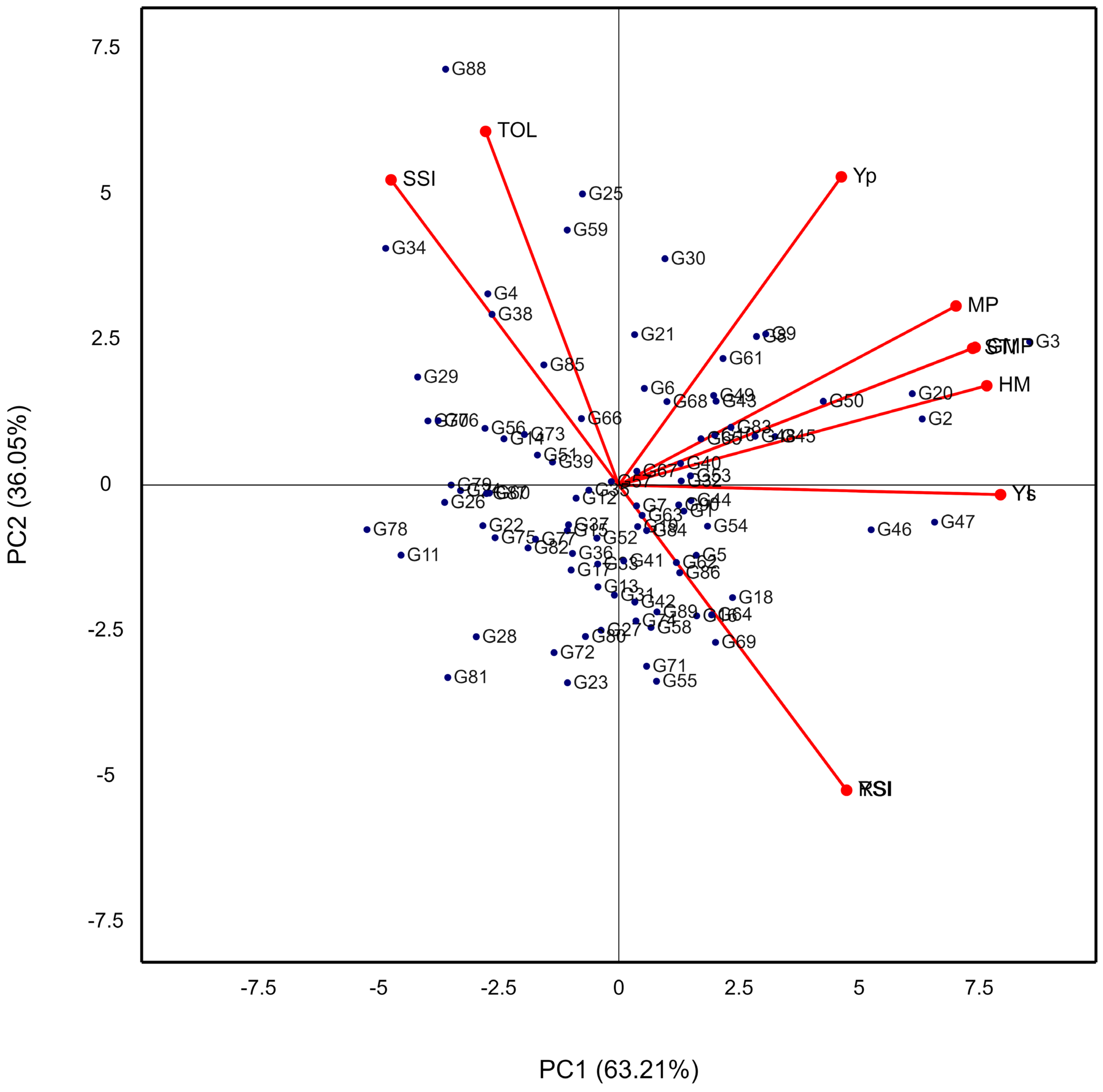

Supplement: Supplementary file 9 — APPENDIX S9. Rendered principal components analysis–based biplot based on the correlation matrix of Yp, Ys, and nine tolerance and susceptibility indices calculated using iPASTIC software for Data Set 1. [file APS3-7-e11278-s009.pdf]
