# Supplementary material for: iPASTIC: An online toolkit to estimate plant abiotic stress indices
Source: Appl Plant Sci. 2019 Jul 17;7(7):e11278. doi: 10.1002/aps3.11278 (PMC6636621; doi:10.1002/aps3.11278)
Supplement: Supplementary file 10 — APPENDIX S10. Yield performance of nine wheat genotypes under control (Yp) and saline (Ys) conditions along with the relative change (RC) due to stress and tolerance and susceptibility indices calculated using iPASTIC software for Data Set 2. [file APS3-7-e11278-s010.docx]

**APPENDIX S10.** Yield performance of nine wheat genotypes under control (Yp) and saline (Ys) conditions along with the relative change (RC) due to stress and tolerance and susceptibility indices calculated using *i*PASTIC software for Data Set 2.^a^

| **Genotype label** | **Yp** | **Ys** | **RC** | **TOL** | **MP** | **GMP** | **HM** | **SSI** | **STI** | **YI** | **YSI** | **RSI** |
| --- | --- | --- | --- | --- | --- | --- | --- | --- | --- | --- | --- | --- |
| *Triticum aestivum* | 160.00 | 80.00 | 50.00 | 80.00 | 120.00 | 113.14 | 106.67 | 1.03 | 1.02 | 1.38 | 0.50 | 0.97 |
| *T. durum* | 160.00 | 90.00 | 43.75 | 70.00 | 125.00 | 120.00 | 115.20 | 0.90 | 1.14 | 1.56 | 0.56 | 1.09 |
| *T. urartu* | 200.00 | 70.00 | 65.00 | 130.00 | 135.00 | 118.32 | 103.70 | 1.34 | 1.11 | 1.21 | 0.35 | 0.68 |
| *T. boeoticum* | 90.00 | 50.00 | 44.44 | 40.00 | 70.00 | 67.08 | 64.29 | 0.92 | 0.36 | 0.87 | 0.56 | 1.08 |
| *Aegilops tauschii* | 60.00 | 40.00 | 33.33 | 20.00 | 50.00 | 48.99 | 48.00 | 0.69 | 0.19 | 0.69 | 0.67 | 1.29 |
| *Ae. neglecta* | 70.00 | 60.00 | 14.29 | 10.00 | 65.00 | 64.81 | 64.62 | 0.29 | 0.33 | 1.04 | 0.86 | 1.66 |
| *Ae. triuncialis* | 110.00 | 50.00 | 54.55 | 60.00 | 80.00 | 74.16 | 68.75 | 1.12 | 0.44 | 0.87 | 0.45 | 0.88 |
| *Ae. crassa* | 40.00 | 20.00 | 50.00 | 20.00 | 30.00 | 28.28 | 26.67 | 1.03 | 0.06 | 0.35 | 0.50 | 0.97 |
| *Ae. caudata* | 120.00 | 60.00 | 50.00 | 60.00 | 90.00 | 84.85 | 80.00 | 1.03 | 0.57 | 1.04 | 0.50 | 0.97 |

^a^ See Table 1 for definitions of indices.
