# Supplementary material for: iPASTIC: An online toolkit to estimate plant abiotic stress indices
Source: Appl Plant Sci. 2019 Jul 17;7(7):e11278. doi: 10.1002/aps3.11278 (PMC6636621; doi:10.1002/aps3.11278)
Supplement: Supplementary file 11 — APPENDIX S11. Yield performance rankings of nine wheat genotypes under control (Yp) and saline (Ys) conditions along with the calculated tolerance and susceptibility indices using iPASTIC software for Data Set 2. [file APS3-7-e11278-s011.docx]

**APPENDIX S11.** Yield performance rankings of nine wheat genotypes under control (Yp) and saline (Ys) conditions along with the calculated tolerance and susceptibility indices using *i*PASTIC software for Data Set 2.^a^

| **Genotype label** | **Yp** | **Ys** | **TOL** | **MP** | **GMP** | **HM** | **SSI** | **STI** | **YI** | **YSI** | **RSI** | **SR** | **AR** | **SD** |
| --- | --- | --- | --- | --- | --- | --- | --- | --- | --- | --- | --- | --- | --- | --- |
| *Triticum aestivum* | 2 | 2 | 8 | 3 | 3 | 2 | 5 | 3 | 2 | 5 | 3 | 38 | 3.45 | 1.86 |
| *T. durum* | 2 | 1 | 7 | 2 | 1 | 1 | 3 | 1 | 1 | 3 | 7 | 29 | 2.64 | 2.29 |
| *T. urartu* | 1 | 3 | 9 | 1 | 2 | 3 | 9 | 2 | 3 | 9 | 1 | 43 | 3.91 | 3.36 |
| *T. boeoticum* | 6 | 6 | 4 | 6 | 6 | 7 | 4 | 6 | 6 | 4 | 6 | 61 | 5.55 | 1.04 |
| *Aegilops tauschii* | 8 | 8 | 2 | 8 | 8 | 8 | 2 | 8 | 8 | 2 | 8 | 70 | 6.36 | 2.80 |
| *Ae. neglecta* | 7 | 4 | 1 | 7 | 7 | 6 | 1 | 7 | 4 | 1 | 9 | 54 | 4.91 | 2.88 |
| *Ae. triuncialis* | 5 | 6 | 5 | 5 | 5 | 5 | 8 | 5 | 6 | 8 | 2 | 60 | 5.45 | 1.63 |
| *Ae. crassa* | 9 | 9 | 2 | 9 | 9 | 9 | 5 | 9 | 9 | 5 | 3 | 78 | 7.09 | 2.77 |
| *Ae. caudata* | 4 | 4 | 5 | 4 | 4 | 4 | 5 | 4 | 4 | 5 | 3 | 46 | 4.18 | 0.60 |

*Note:* ASR = average of sum of ranks; SD = standard deviation of ranks; SR = sum of ranks; Y = yield.

^a^ See Table 1 for definitions of indices.
