# Supplementary material for: iPASTIC: An online toolkit to estimate plant abiotic stress indices
Source: Appl Plant Sci. 2019 Jul 17;7(7):e11278. doi: 10.1002/aps3.11278 (PMC6636621; doi:10.1002/aps3.11278)

APPENDIX S13. Rendered three-dimensional plot based on the STI index and yield performance (Yp and Ys) of the 90 wheat genotypes and accessions in Data Set 2.

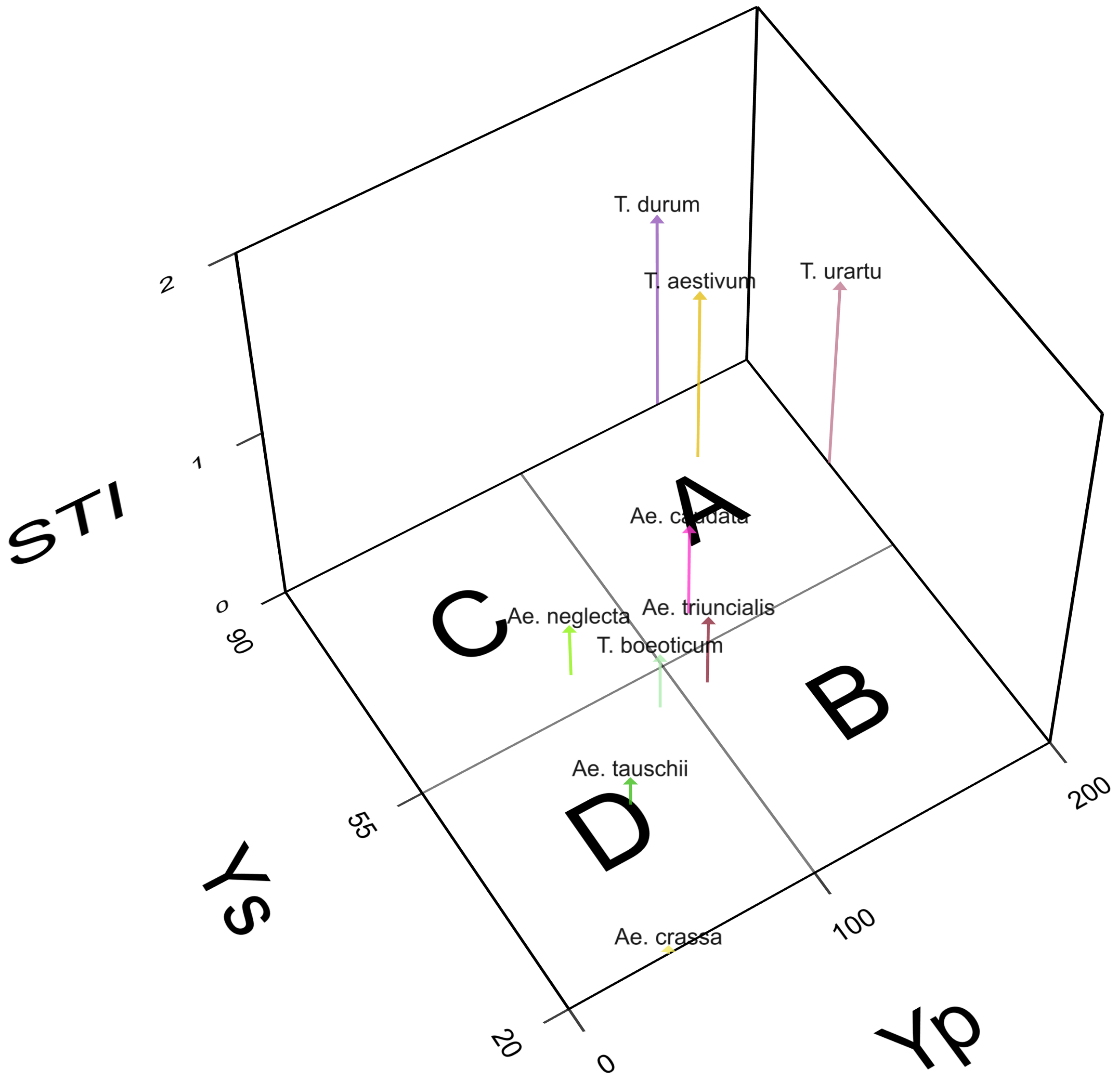

Supplement: Supplementary file 13 — APPENDIX 13. Rendered three‐dimensional plot based on the STI index and yield performance (Yp and Ys) of the 90 wheat genotypes and accessions in Data Set 2. [file APS3-7-e11278-s013.pdf]
