# Supplementary material for: iPASTIC: An online toolkit to estimate plant abiotic stress indices
Source: Appl Plant Sci. 2019 Jul 17;7(7):e11278. doi: 10.1002/aps3.11278 (PMC6636621; doi:10.1002/aps3.11278)

Pour-Aboughadareh et al.—Applications in Plant Sciences 2019 7(7)—Data Supplement S14
DOI

APPENDIX S14. Rendered principal components analysis–based biplot based on the correlation matrix of Yp, Ys, and nine tolerance and susceptibility indices calculated using *i*PASTIC software for Data Set 2.

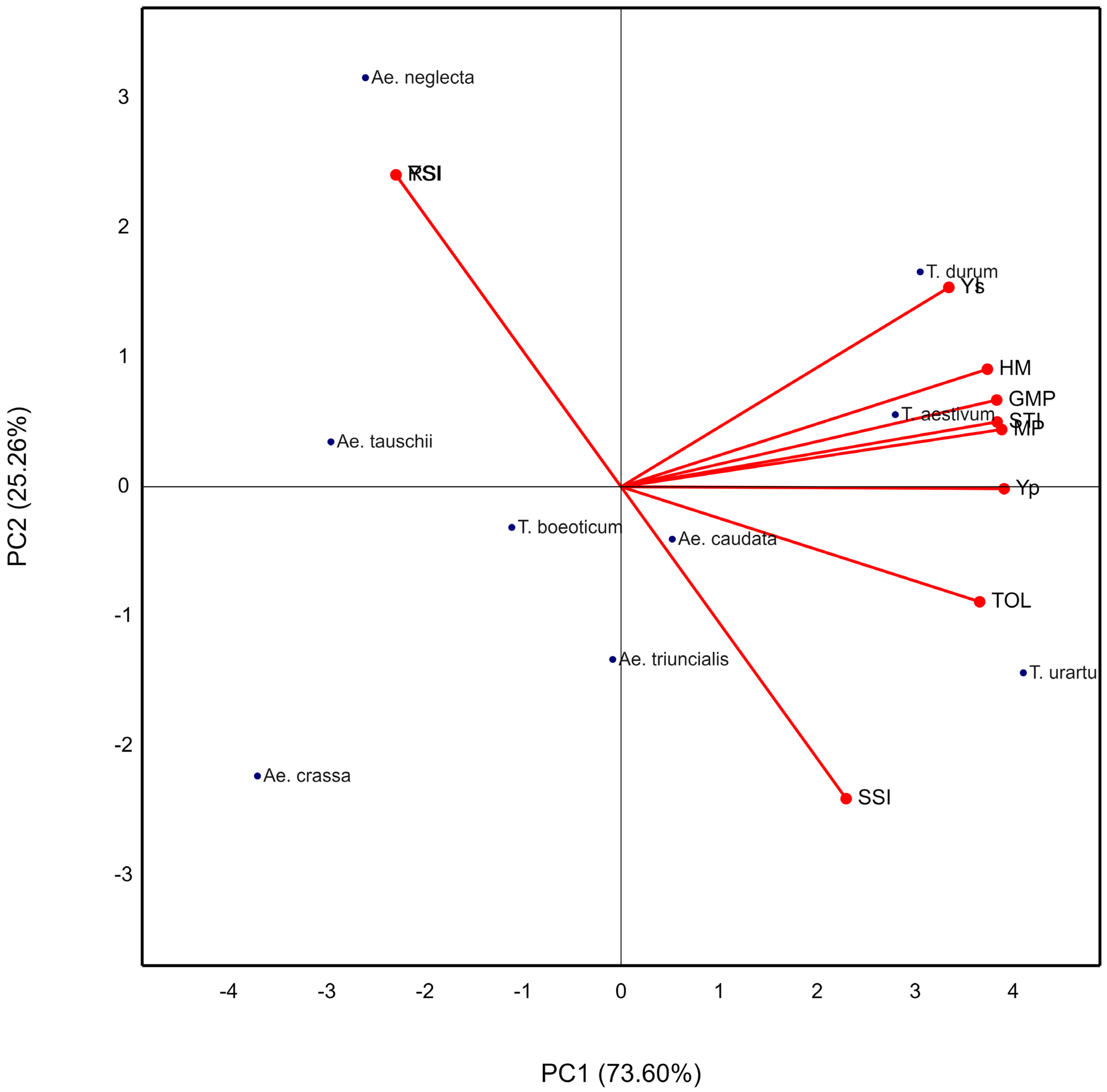

Supplement: Supplementary file 14 — APPENDIX 14. Rendered principal components analysis–based biplot based on the correlation matrix of Yp, Ys, and nine tolerance and susceptibility indices calculated using iPASTIC software for Data Set 2. [file APS3-7-e11278-s014.pdf]
